# Dietary carbohydrate sources differently prime the microbial ecosystem but not the epithelial gene expression profile along the complete gut of young calves

**DOI:** 10.1186/s42523-024-00297-5

**Published:** 2024-03-13

**Authors:** Thomas Hartinger, Cátia Pacífico, Arife Sener-Aydemir, Gregor Poier, Susanne Kreuzer-Redmer, Georg Terler, Fenja Klevenhusen, Qendrim Zebeli

**Affiliations:** 1 Department for Farm Animals and Veterinary Public Health, Institute of Animal Nutrition and Functional Plant Compounds, University of Veterinary Medicine Vienna, Vienna, Austria; 2 Biome Diagnostics GmbH, Vienna, Austria; 3 Unit of Food Hygiene and Technology, Institute of Food Safety, Food Technology and Veterinary Public Health, University of Veterinary Medicine Vienna, Vienna, Austria; 4 Institute of Livestock Research, Agricultural Research and Education Centre Raumberg-Gumpenstein, Irdning-Donnersbachtal, Austria; 5 Department of Environmentally Sustainable Animal Nutrition, University of Kassel, Kassel, Germany

**Keywords:** Concentrate, Gene expression, Gut, Hay quality, Microbiome, Rearing, Ruminant, Starch, 16S rRNA sequencing

## Abstract

**Background:**

Recent data indicated similar growth performance of young calves fed solely high-quality hay instead of a starter diet based on starchy ingredients. Yet, providing exclusively such distinct carbohydrate sources during early life might specifically prime the microbiota and gene expression along the gut of young calves, which remains to be explored. We investigated the effects of starter diets differing in carbohydrate composition, that is medium- or high-quality hay and without or with 70% concentrate supplementation (on fresh matter basis), across the gastrointestinal tract (GIT) of weaned Holstein calves (100 ± 4 days of age) using 16 S rRNA gene sequencing and analyses of short-chain fatty acids and host epithelial gene expressions.

**Results:**

The concentrate supplementation drastically decreased microbial diversity throughout the gut, which was also true to a much lesser extent for high-quality hay when compared to medium-quality hay in the foregut. Similarly, the factor concentrate strongly shaped the diet-associated common core microbiota, which was substantially more uniform along the gut with concentrate supplementation. The fermentation profile shifted towards less acetate but more propionate with concentrate supplementation in almost all gut sections, corresponding with higher abundances of starch-utilizing bacteria, while major fibrolytic clusters declined. Noteworthy, the n-butyrate proportion decreased in the rumen and increased in the colon with concentrate, showing an opposite, gut site-dependent effect. Both dietary factors modestly influenced the host epithelial gene expression.

**Conclusions:**

Concentrate supplementation clearly primed the microbial ecosystem on a starch-targeted fermentation with characteristic genera occupying this niche along the entire GIT of calves, whereas the microbial differentiation due to hay quality was less distinct. Overall, changes in the microbial ecosystem were only marginally reflected in the targeted transcriptional profile of the host epithelium.

**Supplementary Information:**

The online version contains supplementary material available at 10.1186/s42523-024-00297-5.

## Background

Young dairy calves are typically fed milk or milk replacers and supplemented with starchy starter diets to ensure rapid growth during early life. New research has suggested the use of solely high-quality hay, meaning hay rich in nutrients such as sugars and relatively low in fiber without any starch, as an alternative to starchy starter diets for dairy calves. These experiments have demonstrated similar growth performance of calves when compared to calves fed concentrate-dominated diets with medium-quality hay [1]. Besides potential economic and ecological benefits due to savings on concentrate, important animal gut health aspects emphasize the advantageousness of such pure hay-based starter diets for calves: Feeding only high-quality hay instead of hay plus concentrate increased the rumination activity [2] as well as it beneficially imprinted the rumen around weaning, i.e., the promotion of a more diverse microbiota with higher abundances of fibrolytic key bacteria [3], which was also reflected in higher ruminal n-butyrate concentrations [2]. Interestingly, feeding hay-based starter diets without any starch did not only cause higher ruminal n-butyrate concentrations but also improved the ketogenesis and cholesterogenesis as evidenced by elevated levels of beta hydroxybutyrate (BHB) and cortisol in blood serum [1, 2]. Since the liver as the main place of ketone body biosynthesis was presumably not causative for these distinct BHB levels [1], higher ketogenesis-associated gene expression in the rumen epithelium may explain this increase but awaits to be elucidated.

Apart from the rumen, a clear impact of such distinct starter diets on the microbial ecosystem, meaning the microbial composition and the fermentation profile, in the lower GIT of the calves appears very likely, too. Indeed, research provides clear evidence for the shaping force of the diet on the small intestinal and hindgut microbiome of young ruminants [4–6] and consequences may as well be observed in the host epithelium that is directly interacting with the gut microbiome [7]. For instance, Holstein calves receiving milk replacer plus a starchy solid feed showed upregulations of several tight junction genes in the small intestine compared to calves solely fed with milk replacer, while the solid starter also altered the bacterial diversity and calves’ regulation of secretory defense molecules [4].Consequently, the present study aimed to establish the impact of feeding starter diets with contrasting dietary carbohydrate sources on the microbial communities, fermentation profiles and specific host gene expressions along the complete gut of calves. More precisely, starter diets differed in two dietary factors. First, in hay quality, i.e., either medium-quality hay that was low in water-soluble carbohydrates (WSC; 12.4% in dry matter (DM)) but high in neutral detergent fiber (NDF; 52.2% in DM) or high-quality hay that was rich in sugars (20.5% in DM) and relatively low in NDF (45.5% in DM). And as second factor, without or with the replacement of hay by 70% starchy grain supplementation (fresh matter basis), leading to 0% or 40% starch inclusion on a DM basis, respectively. Provided that recognized effects of starter diets on the calves’ gut ecosystem are persistent, the present study may ultimately contribute to the design of calf feeding regimes that could support the rearing of productive and healthy dairy cattle. We hypothesized a microbiota composition primed on starch utilization as well as a fermentation profile shifted towards glucogenic precursors in response to concentrate inclusion along the entire GIT, which would be also reflected in the host epithelial transcription profile. For pure hay feeding, we in particular expected higher acetate and n-butyrate concentrations in all gut sections, especially in the rumen, leading to a promoted expression of genes involved in ketogenesis.

## Results

### Microbial communities along the gastrointestinal tract

The general dataset characteristics revealed an average read count of 31,002.0 for the reticulum, 31,919.5 for rumen liquid, 30,676.9 for rumen solid, 32,591.3 for abomasum, 33,249.6 for duodenum, 37,192.9 for jejunum and 28,088.6 for the colon. The total read counts and percentage of total read counts at the phylum divided by gut location, are given in Supplementary Table 1. In all gut locations, Firmicutes, Bacteroidetes, Proteobacteria, and Actinobacteria were the four most abundant phyla, accounting for 85.4 to 92.9% of the microbial communities. At the genus level, *Succinivibrionaceae* UCG − 001, *Prevotella* 1, *Prevotella 7*, *Sharpea* and *Acetitomaculum* were the top five most abundant genera, but showed divergences across the GIT, which was particularly true for *Succinivibrionaceae* UCG − 001, *Prevotella* 1 and *Prevotella 7* (Supplementary Fig. 1).

### Diversity

Regarding alpha diversity, the numbers of observed sub-operational taxonomic unit (sOTU) did only tendentially differ along the GIT of calves (*P* = 0.07), whereas the gut location affected the Shannon (*P* < 0.01), InvSimpson (*P* = 0.01) and Fisher’s alpha diversity metrics (*P* = 0.01; Table 1). The highest diversity values were observed in the colon, while the other gut sites showed lower indices, especially with regard to InvSimpson index. Table 2 presents the effects of hay quality, concentrate supplementation and their interaction on the alpha diversity indices separated by gut site. We observed interactions of hay quality and concentrate supplementation for several indices in the reticulum, rumen solid, duodenum or colon that all had the same pattern (each *P* ≤ 0.04), i.e., highest values for MQH (100% medium-quality hay), followed by HQH (100% medium-quality hay) and lowest values for MQH + C (30% medium-quality hay and 70% concentrate; on fresh matter basis) and HQH + C (30% high-quality hay and 70% concentrate; on fresh matter basis). The supplementation with concentrate reduced all alpha diversity indices in all gut sites (each *P* < 0.01), except for the InvSimpson index in the jejunum (*P* = 0.10). Likewise, feeding HQH instead of MQH reduced the alpha diversity indices, although the impact of hay quality was less frequent and only significant for the reticulum (all indices with each *P* ≤ 0.05), rumen solid (only InvSimpson index with *P* < 0.01 and Shannon with *P* = 0.09) and as a trend for Fisher’s index in the colon (*P* = 0.06).

**Fig. 1:**
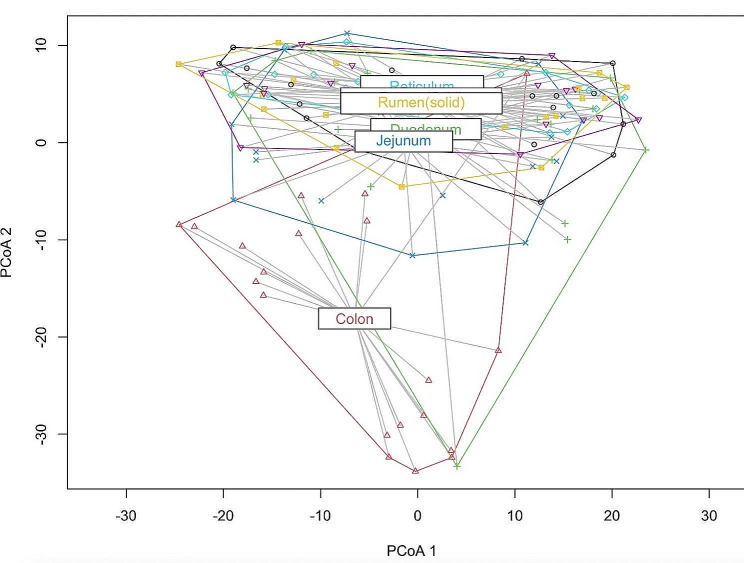
Principal Coordinates Analysis (PCoA) plot using clr-transformed data in Aitchison metrics visualizing ordination centroids and dispersions for gut locations

**Fig. 2:**
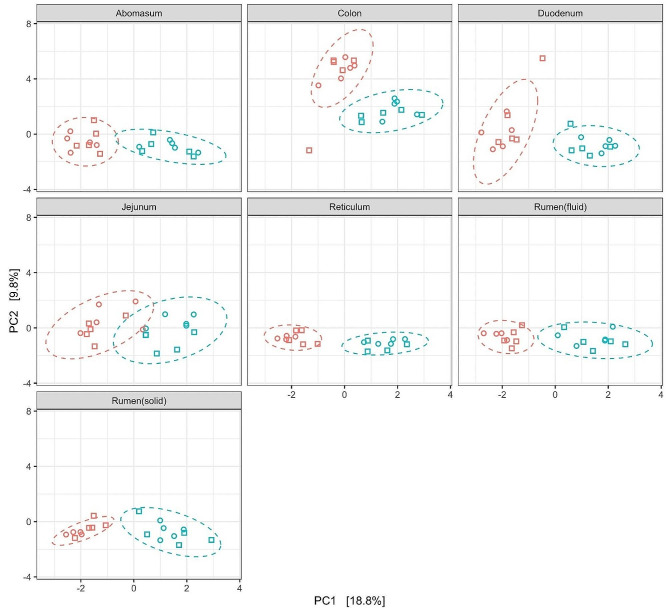
Changes in prokaryotic community composition visualized as a Principal Coordinates Analysis (PCoA) using clr-transformed data in Aitchison metrics. Different shapes illustrate hay quality, i.e., medium quality (circle) or high quality (square), and different colors indicate concentrate supplementation, i.e., without (red) or with 70% concentrate supplementation (on fresh matter basis; blue). The percentage of variation explained is indicated on the respective axes and ellipses illustrate the 95% confidence intervals for concentrate supplementation

With regard to beta diversity, the PCoA analysis displayed a segregated cluster of colon-derived samples from all other gut sites (for each pairwise comparison *P* ≤ 0.02; Fig. 1). On gut site-specific level, concentrate supplementation led to a separation along PCoA axis 1 for all gut locations (each *P* < 0.01), whereas hay quality led to no clustering of the data (each *P* > 0.10; Fig. 2).

**Table 1:** Differences in alpha diversity indices between gastrointestinal tract sections of calves

	Reticulum	Rumen liquid	Rumen solid	Abomasum	Duodenum	Jejunum	Colon	SEM^1^	P-value
Observed sOTU^2^	748	768	781	809	784	781	865	60.4	0.07
Shannon	4.18^bc^	4.07^bc^	4.23^b^	4.16^bc^	4.14^bc^	3.87^c^	4.87^a^	0.21	< 0.01
InvSimpson	27.8^b^	23.9^b^	32.5^ab^	24.9^b^	24.8^b^	29.7^ab^	53.1^a^	7.66	0.01
Fisher’s	145^b^	149^b^	153^ab^	156^ab^	150^b^	147^b^	178^a^	15.0	0.01

^1^Standard error of the mean

^2^Sub-operational taxonomic unit

**Table 2:** Effects of hay quality and concentrate supplementation on alpha diversity indices along the gastrointestinal tract of calves

	Treatment						*P*-values				
Item	MQH^1^	HQH^2^	MQH + C^3^	HQH + C^4^		SEM^5^		Hay quality	Concentrate	Hay quality × Concentrate	
Reticulum											
Observed sOTU^6^	1109^a^	861^b^	500^c^	502^c^		51.3		0.02	< 0.01	0.02	
Shannon	5.31	4.80	3.27	3.23		0.14		0.05	< 0.01	0.10	
InvSimpson	57.8^a^	33.1^b^	11.5^c^	9.33^c^		4.94		0.01	< 0.01	0.03	
Fisher’s	238^a^	171^b^	84.4^c^	83.6^c^		13.0		0.02	< 0.01	0.02	
Rumen liquid											
Observed sOTU	1051	895	570	522		74.0		0.15	< 0.01	0.44	
Shannon	4.99	4.80	3.33	3.21		0.17		0.31	< 0.01	0.81	
InvSimpson	33.4	32.1	11.7	9.72		5.27		0.74	< 0.01	0.94	
Fisher’s	221	178	98.2	89.8		17.4		0.13	< 0.01	0.30	
Rumen solid											
Observed sOTU	1059	880	599	550		74.0		0.12	< 0.01	0.36	
Shannon	5.33	4.88	3.37	3.33		0.15		0.09	< 0.01	0.16	
InvSimpson	65.1^a^	39.4^b^	10.9^c^	10.4^c^		3.47		< 0.01	< 0.01	< 0.01	
Fisher’s	226	174	106	98.6		18.0		0.09	< 0.01	0.20	
Abomasum											
Observed sOTU	1078	895	659	605		80.2		0.16	< 0.01	0.43	
Shannon	4.95	4.80	3.42	3.46		0.21		0.81	< 0.01	0.66	
InvSimpson	43.8	33.5	10.8	11.4		6.05		0.44	< 0.01	0.38	
Fisher’s	220	178	120	107		18.3		0.15	< 0.01	0.45	
Duodenum											
Observed sOTU	1062^a^	881^b^	521^c^	672^c^		55.4		0.79	< 0.01	0.01	
Shannon	5.15	4.67	3.30	3.42		0.20		0.37	< 0.01	0.15	
InvSimpson	45.4	31.7	11.4	10.7		18.7		0.33	< 0.01	0.77	
Fisher’s	215	176	88.8	119		13.8		0.76	< 0.01	0.20	
Jejunum											
Observed sOTU	1058	896	581	588		73.3		0.31	< 0.01	0.27	
Shannon	5.09	4.67	2.64	3.09		0.27		0.96	< 0.01	0.13	
InvSimpson	75.2	28.6	5.81	9.00		25.7		0.41	0.10	0.35	
Fisher’s	217	175	97.5	99.7		32.6		0.41	< 0.01	0.33	
Colon											
Observed sOTU	1225	980	617	639		72.4		0.14	< 0.01	0.08	
Shannon	5.70	5.33	4.21	4.22		0.17		0.31	< 0.01	0.28	
InvSimpson	93.8	65.4	25.6	27.6		13.5		0.34	< 0.01	0.28	
Fisher’s	279^a^	204^b^	112^c^	117^c^		17.5		0.06	< 0.01	0.04	

^1^Medium-quality hay without concentrate supplementation

^2^High-quality hay without concentrate supplementation

^3^Medium-quality hay with 70% concentrate supplementation (on fresh matter basis)

^4^High-quality hay with 70% concentrate supplementation (on fresh matter basis)

^5^Standard error of the mean

^6^Sub-operational taxonomic unit

Numbers within a row with different superscript letters indicate difference (*P* < 0.05)

### Differential abundance

In the reticulum, concentrate supplementation affected the abundance of 72 genera and hay quality that of one genus. *Ruminiclostridium* 6, *Butyrivibrio* 2, an uncultured bacterium of the order *Bacteroidales*, *Lachnospiraceae* XPB1014 group and *Saccharofermentans* were the genera that most decreased by concentrate supplementation (coefficient <-4.50, *q* < 0.05), whereas *Mitsuokella*, *Sharpea*, *Acidaminococcus*, *Succinivibrionaceae* UCG-001 and *Prevotella 7* were most increased (coefficient > 4.00, *q* < 0.05). Only *Shuttleworthia* was increased by HQH when compared to MQH (coefficient = 2.16, *q* < 0.05). For solid and liquid rumen content, 76 genera were affected by concentrate supplementation (coefficient > ± 2.00, *q* < 0.05) and two genera, i.e., *Lachnospiraceae* FCS020 group (coefficient = 2.08, *q* < 0.05) and *Ruminococcaceae* UCG-011 (coefficient = 2.83, *q* < 0.05) were less abundant with HQH than with MQH. Regarding concentrate supplementation, an uncultured bacterium of *Bacteroidales*, *Coprococcus* 2, *Papillibacter*, *Ruminococcaceae* UCG-011 and *Butyrivibrio*_2 (coefficient <-4.70, *q* < 0.05) as well as *Butyrivibrio* 2, *Anaerorhabdus furcosa* group, *Ruminiclostridium* 6, *Lachnospiraceae* XPB1014 group and *Veillonellaceae* UCG-001 (coefficient <-4.45, *q* < 0.05) were most decreased by concentrate supplementation in rumen liquid and rumen solid, respectively. In contrast *Acidaminococcus*, *Megasphaera*, *Sharpea*, *Prevotella 7* and *Succinivibrionaceae* UCG-001 (coefficient > 4.00, *q* < 0.05) as well as *Sharpea*, *Mitsuokella*, *Acidaminococcus*, *Succinivibrionaceae* UCG-001 and *Prevotella 7* (coefficient > 4.20, *q* < 0.05) were most increased by concentrate supplementation in rumen liquid and rumen solid, respectively. In the abomasum, 71 genera were differently abundant due to concentrate supplementation, which increased *Acidaminococcus*, *Sharpea*, *Mitsuokella*, *Succinivibrionaceae* UCG-001 and *Prevotella 7* the most (coefficient > 4.00, *q* < 0.05), while *Papillibacter*, *Coprococcus* 1 and *Saccharofermentans* were most decreased (coefficient <-4.00, *q* < 0.05). Hay quality, however, did not affect the microbial abundances in the abomasum, which was also true for the duodenum. Again, concentrate supplementation changed the abundance of 77 genera with *Papillibacter*, *Eubacterium hallii* group, *Ruminiclostridium*_9, *Ruminococcaceae* UCG-013, *Coprococcus* 1 and *Christensenellaceae* R7 group (coefficient <-4.00, *q* < 0.05) as well as *Megasphaera*, *Sharpea*, *Succinivibrionaceae* UCG-001, *Mitsuokella*, *Acidaminococcus* and *Prevotella 7* (coefficient > 4.00, *q* < 0.05) being most decreased and increased in the duodenum, respectively. Regarding the jejunum, *Coprococcus* 2 was less present with medium- than with high-quality hay feeding (coefficient =−2.50, *q* < 0.05), whereas *Bacillus* was higher abundant with medium- than with high-quality hay (coefficient = 3.11, *q* < 0.05). Likewise, 45 genera were differently abundant due to concentrate supplementation in the jejunum: *Megasphaera*, *Mitsuokella*, *Pseudoramibacter* and *Sharpea* were higher with concentrate supplementation than without (coefficient > 3.30, *q* < 0.05), whereas *Eubacterium hallii* group, Family XIII UCG-001, *Ruminiclostridium* 9, *Coprococcus* 1 and *Ruminococcaceae* UCG-013 were most decreased by concentrate supplementation (coefficient <-4.50, *q* < 0.05). *Collinsella* was the only genus in the colon that was increased by high-quality hay when compared to medium-quality hay (coefficient =−2.66, *q* < 0.05), while three uncultured bacteria belonging to *Izimaplasmatales*, *Gastranaerophilales* or *Bacteroidales* were decreased with high-quality hay feeding in the colon (coefficient > 2.03, *q* < 0.05). The concentrate supplementation changed the abundance of 71 genera with *Lachnoclostridium* 10, *Ruminiclostridium* and *Ruminococcaceae* UCG-10 constituting the top 3 decreased genera (coefficient <-5.50, *q* < 0.05), while ND3007 group belonging to *Lachnospiraceae*, *Faecalibacterium* and *Prevotella 9* represented the top 3 increased genera (coefficient > 4.90, *q* < 0.05). All differential abundances for each gut location, including exact coefficients and *q*-values, are presented in Supplementary Table 2.

### Diet-associated common core microbiota

**Fig. 3:**
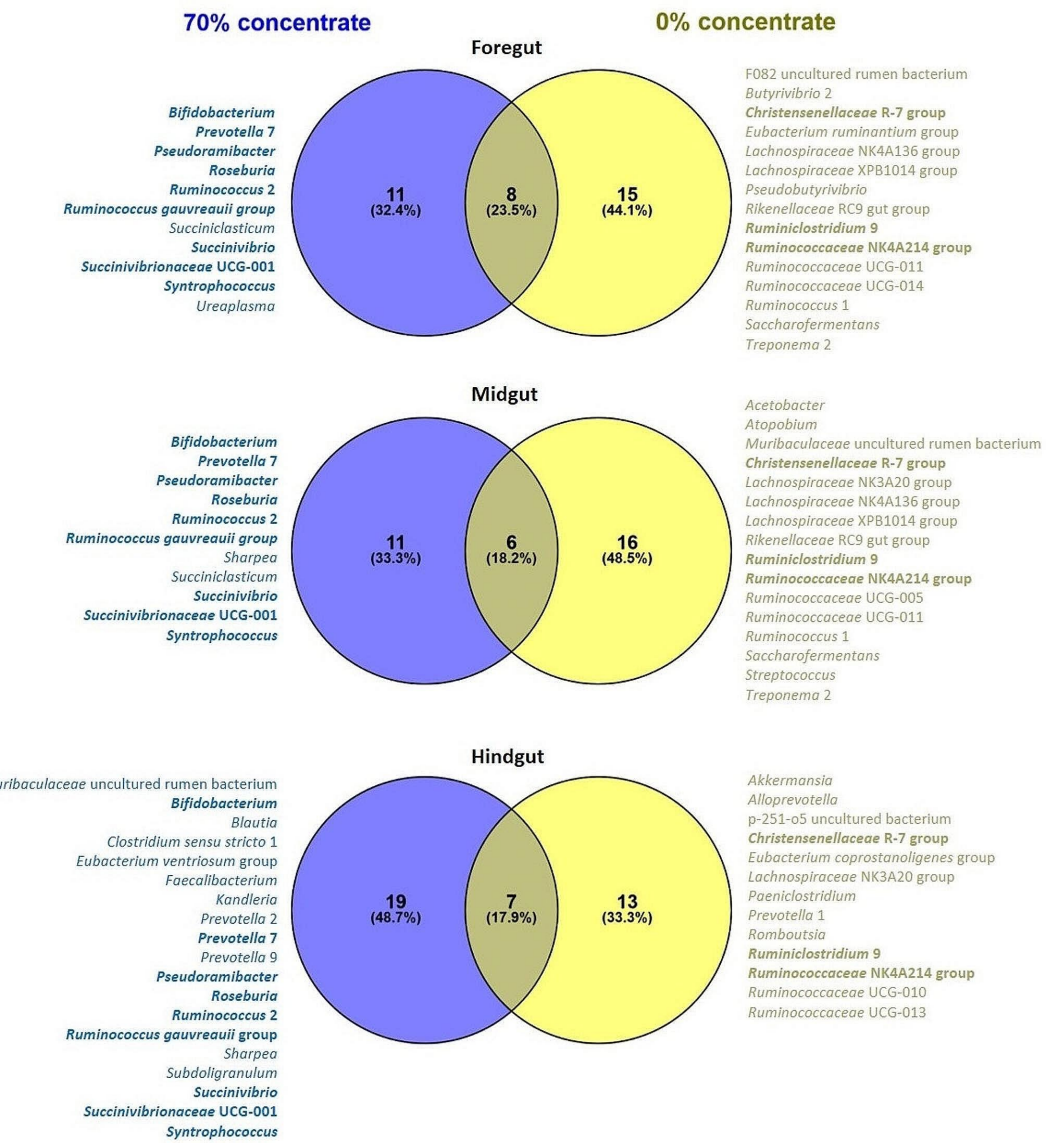
Diet-associated common core microbiota at genus level of the three major gut regions fore-, mid- and hindgut in calves fed starter diets without or with 70% concentrate supplementation (on fresh matter basis). Laterally listed genera are exclusively present for the respective starter diet and visualized in bold when present in all three major gut regions

Regarding the diet-associated common core microbiota (CCM) in the digesta, calves fed starter diets with or without concentrate supplementation shared 23.5% (eight genera), 18.2% (six genera) and 17.9% (seven genera) of CCM genera in the fore, mid- and hindgut, respectively (Fig. 3). The majority of CCM-genera, however, depended on the factor concentrate and were exclusively present in one of the starter diets. When calves received no concentrate, three CCM-genera were shared between all major gut regions, i.e., *Christensenellaceae* R-7 group, *Ruminiclostridium* 9 and *Ruminococcaceae* NK4A214 group, whereas nine CCM-genera were shared between the major gut regions in calves with concentrate supplementation, i.e., *Bifidobacterium*, *Prevotella* 7, *Pseudoramibacter*, *Roseburia*, *Ruminococcus* 2, *Ruminococcus gauvreauii* group, *Succinivibrio*, *Succinivibrionaceae* UCG-001 and *Syntrophococcus*. Without considering whether CCM-genera were exclusive for a starter diet, concentrate supplementation as well resulted in a smaller overall CCM than without, meaning 32 vs. 37 CCM-genera (Supplementary Fig. 2). Again, a higher proportion of CCM-genera was shared for all major gut regions when calves received starter diets with concentrate compared to starter diets without concentrate, i.e., 43.8% vs. 27.0%.

**Fig. 4:**
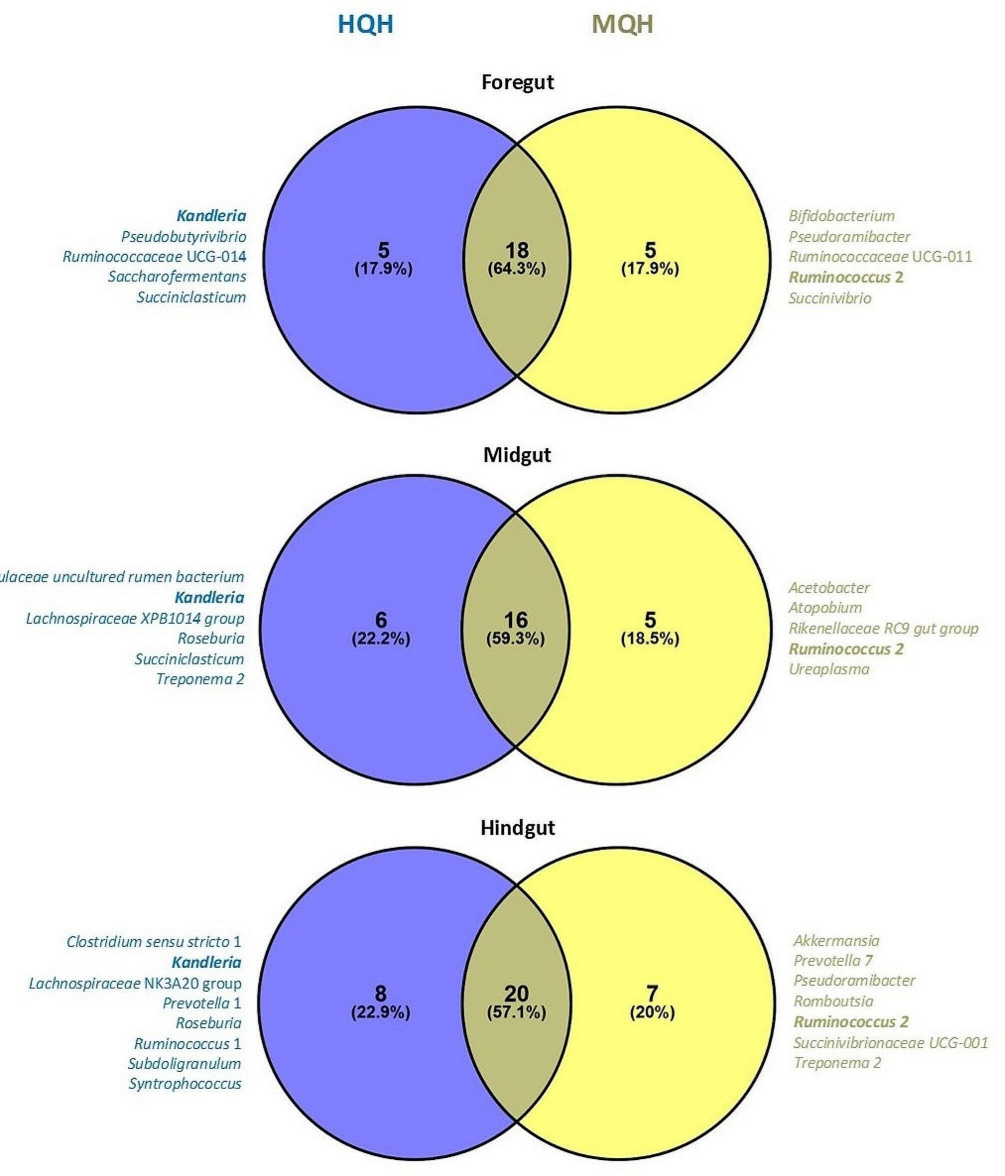
Diet-associated common core microbiota at genus level of the three major gut regions fore-, mid- and hindgut in calves fed starter diets with high- (HQH) or medium-quality hay (MQH). Laterally listed genera are exclusively present for the respective starter diet and visualized in bold when present in all three major gut regions

The factor hay quality resulted in a high number of shared genera between high- and medium-quality hay in all three major gut regions, i.e., 64.3% (18 genera), 59.3% (16 genera) and 57.1% (20 genera) for the fore-, mid- and hindgut, respectively (Fig. 4). Five CCM-genera were exclusive for high- and medium quality hay each in the foregut, while six vs. five and eight vs. seven CCM-genera were exclusive for high- vs. medium-quality hay feeding in the mid- and hindgut, respectively. Among those exclusively present CCM-genera, *Kandleria* and *Ruminococcus* 2 were part of the CCM in all major gut regions for high- and medium-quality hay, respectively. Without considering whether CCM-genera were exclusive for one hay quality type, feeding high-quality hay led to an overall CCM consisting of 40 genera, while medium-quality hay feeding resulted in an overall CCM of 39 genera (Supplementary Fig. 3). Besides, a higher proportion of CCM-genera were shared for all major gut regions when calves received medium-quality hay instead of high-quality hay, i.e., 33.3% vs. 27.5%.

### Predicted function

The analysis of predicted functional profiles revealed the differential abundance of 319 pathways across the entire GIT, which are listed in Supplementary Table 3, including all coefficients, standard deviations and *q*-values. The majority (62%) of differently abundant pathways were found in the foregut and 96% of total differently abundant pathways were affected by concentrate supplementation. For instance, concentrate supplementation upregulated the pathway associated with glucose and glucose-1-phosphate degradation (GLUCOSE1PMETAB-PWY; coefficients ≥ 3.40, each *q* < 0.01) from the reticulum until the duodenum, whereas it downregulated predicted pathways associated with acetate and butyrate production (CENTFERM-PWY, P163-PWY, PWY-5676, CODH-PWY; coefficients ≤ −2.10, each *q* < 0.01) along the entire GIT, except the jejunum. Regarding hay quality, abundances of 13 pathways were differently abundant, such as an upregulation of pathways associated with the degradation of aromatic molecules (CATECHOL-ORTHO-CLEAVAGE-PWY, PROTOCATECHUATE-ORTHO-CLEAVAGE-PWY, PWY-5417, PWY-5431; coefficients ≥ 2.39, each *q* ≤ 0.03) in the reticulum when feeding medium- instead of high-quality hay.

### Short-chain fatty acid profiles along the gastrointestinal tract

As shown in Table 3, the gut location had a clear impact on the short-chain fatty acid (SCFA) profiles with total SCFA being highest in the rumen, followed by the reticulum and colon, and abomasum, duodenum and jejunum with the lowest concentrations (*P* < 0.01). Regarding the individual SCFA, except for iso-butyrate (*P* > 0.10), all SCFA proportions were affected by gut location (each *P* < 0.01): for acetate as the most abundant SCFA (≥ 60% for all gut locations), proportions were lower in reticulum and rumen compared to lower gut locations. Propionate was the second most abundant SCFA and proportionally highest in the foregut, i.e., reticulum, rumen liquid and rumen solid (26.9–28.0%), while especially low in the jejunum (6.25%). Likewise, n-butyrate, n-valerate and iso-valerate proportions were higher in the foregut than in the abomasum and small intestine. For the colon, proportions of n-butyrate and iso-valerate were comparable to the foregut, while n-valerate proportion was similar to abomasum and duodenum.

Due to these substantial differences between the different gastrointestinal sections, dietary treatment effects, meaning the effects of hay quality, concentrate supplementation and their interaction, were analyzed for each gut location separately (Table 4). Both HQH (*P* = 0.05) and concentrate supplementation (*P* < 0.01) increased the total SCFA concentration in the rumen liquid compared to MQH and no concentrate supplementation, respectively. Similarly, HQH increased total SCFA in the colon compared to MQH (*P* < 0.01), whereas concentrate supplementation led to a reduction of total SCFA in this gut location (*P* < 0.01). Though this actually applied to HQH and not truly for MQH. The total SCFA concentrations in the other gut sites were not affected by hay quality, concentrate supplementation or their interaction (each *P* > 0.10). Except for the jejunum and colon, acetate proportion was higher and propionate proportion was lower for MQH than for HQH (*P* ≤ 0.01). However, the propionate proportion in the duodenum was higher in HQH + C than in HQH and MQH, with MQH + C being intermediate, as evidenced by an interaction for this variable (*P* < 0.01). A further interaction was found for n-butyrate proportion in abomasum (*P* = 0.02). In contrast to reticulum and rumen, n-butyrate was lower with MQH compared to HQH feeding, but solely in hay-only diets. Besides, compared to MQH feeding, HQH increased the n-valerate proportions in the reticulum (*P* = 0.04), but decreased the proportions of iso-butyrate (*P* = 0.06) and iso-valerate (*P* = 0.04) in the colon.

Regarding the impact of concentrate supplementation, SCFA proportions were affected in all gut sites, except for the jejunum. The addition of concentrate to the starter diet had a similar effect on all sites in the foregut, i.e., reticulum, rumen liquid and solid rumen content, with higher proportions of propionate (each *P* < 0.01) and n-valerate (each *P* < 0.01), but lower proportions of acetate (*P* ≤ 0.02), n-butyrate (*P* ≤ 0.01), iso-butyrate (*P* ≤ 0.02) and iso-valerate (*P* ≤ 0.02) compared to no concentrate supplementation. In the abomasum, concentrate supplementation similarly shifted the SCFA profile as described for the foregut. As observed for the other gut sites, the acetate proportion also decreased with concentrate supplementation in the duodenum (*P* = 0.02) and colon (*P* < 0.01), i.e., around 11% and 8.6%, respectively. Likewise, concentrate supplementation decreased the colonic proportions of n-valerate, iso-valerate and iso-butyrate, whereas it increased the propionate and n-butyrate proportions (each *P* < 0.01). In contrast, the duodenal n-valerate proportion was higher with concentrate supplementation than without (*P* < 0.01).

**Table 3:** Differences in short-chain fatty acid (SCFA) profiles between gastrointestinal tract sections of calves

	Reticulum	Rumen liquid	Rumen solid	Abomasum	Duodenum	Jejunum	Colon	SEM^1^	P-value
Total SCFA, µmol/g	90.1^b^	135^a^	153^a^	19.1^c^	9.08^c^	8.06^c^	76.3^b^	4.10	< 0.01
Acetate, %	62.0^c^	59.6^c^	59.9^c^	70.2^b^	77.1^b^	91.2^a^	71.8^b^	1.48	< 0.01
Propionate, %	26.9 ^ab^	28.0^a^	27.9^a^	22.5^b^	18.4^bc^	6.25^d^	17.3^c^	1.17	< 0.01
n-Butyrate, %	6.66^a^	7.50^a^	7.36^a^	4.41^b^	2.99^bc^	1.37^c^	7.96^a^	0.52	< 0.01
Iso-Butyrate, %	0.88	0.79	0.78	0.67	0.49	0.98	1.00	0.21	0.60
n-Valerate, %	2.18^ab^	2.86^a^	2.88^a^	1.55^b^	0.78^b^	0.12^c^	1.10^b^	0.26	< 0.01
Iso-Valerate, %	0.92^a^	0.79^a^	0.77^a^	0.48^b^	0.22^bc^	0.02^c^	0.81^a^	0.08	< 0.01

^1^Standard error of the mean

**Table 4:** Effects of hay quality and concentrate supplementation on short-chain fatty acid (SCFA) profiles along the gastrointestinal tract of calves

	Treatment					*P*-values		
Item	MQH^1^	HQH^2^	MQH + C^3^	HQH + C^4^	SEM^5^	Hay quality	Concentrate	Hay quality × Concentrate
Reticulum								
Total SCFA, µmol/g	74.9	106	85.7	94.6	12.8	0.13	0.99	0.38
Acetate, %	72.2	66.6	58.7	50.0	1.62	< 0.01	< 0.01	0.33
Propionate, %	16.7	20.5	32.0	38.6	1.42	< 0.01	< 0.01	0.32
n-Butyrate, %	7.66	9.21	4.78	5.54	0.78	0.14	< 0.01	0.60
Iso-Butyrate, %	1.25	0.92	0.66	0.71	0.13	0.28	0.02	0.16
n-Valerate, %	0.71	1.45	2.82	4.06	0.47	0.04	< 0.01	0.58
Iso-Valerate, %	1.34	1.03	0.69	0.64	0.16	0.27	0.01	0.41
Rumen liquid								
Total SCFA, µmol/g	108	135	145	150	8.23	0.05	< 0.01	0.16
Acetate, %	70.6	65.1	54.2	48.5	1.56	< 0.01	< 0.01	0.92
Propionate, %	17.8	21.2	33.7	39.3	1.62	0.01	< 0.01	0.47
n-Butyrate, %	8.52	9.97	5.87	5.71	1.03	0.51	< 0.01	0.41
Iso-Butyrate, %	0.98	0.84	0.72	0.61	0.09	0.15	0.01	0.83
n-Valerate, %	0.81	1.67	4.18	4.78	0.81	0.34	< 0.01	0.86
Iso-Valerate, %	1.08	0.88	0.71	0.54	0.12	0.14	0.01	0.89
Rumen solid								
Total SCFA, µmol/g	127	161	156	170	12.7	0.06	0.13	0.39
Acetate, %	70.9	65.7	54.7	48.4	1.58	< 0.01	< 0.01	0.72
Propionate, %	17.9	21.1	33.2	39.3	1.55	0.01	< 0.01	0.32
n-Butyrate, %	8.27	9.64	5.87	5.75	1.07	0.53	0.01	0.46
Iso-Butyrate, %	0.97	0.85	0.72	0.59	0.09	0.15	0.01	0.99
n-Valerate, %	0.81	1.65	4.20	4.88	0.87	0.36	< 0.01	0.92
Iso-Valerate, %	0.97	0.87	0.72	0.55	0.12	0.25	0.02	0.72
Abomasum								
Total SCFA, µmol/g	13.7	18.8	27.8	16.0	8.44	0.66	0.48	0.14
Acetate, %	81.1	74.0	65.1	60.8	2.00	0.01	< 0.01	0.49
Propionate, %	13.1	16.8	27.7	32.5	1.32	0.01	< 0.01	0.72
n-Butyrate, %	3.76^b^	6.43^a^	3.87^b^	3.58^b^	0.56	0.05	0.03	0.02
Iso-Butyrate, %	1.17	1.05	0.33	0.11	0.14	0.24	< 0.01	0.75
n-Valerate, %	0.06	0.99	2.48	2.68	0.39	0.17	< 0.01	0.36
Iso-Valerate, %	0.83	0.69	0.28	0.10	0.21	0.46	0.01	0.93
Duodenum								
Total SCFA, µmol/g	7.60	10.9	9.08	8.99	1.49	0.27	0.89	0.24
Acetate, %	87.3	78.1	78.2	64.6	4.34	0.01	0.02	0.60
Propionate, %	9.60^b^	15.4^b^	18.3^ab^	30.3^a^	5.14	< 0.01	0.12	< 0.01
n-Butyrate, %	2.50	4.52	2.01	3.23	1.00	0.11	0.36	0.68
Iso-Butyrate, %	0.31	1.46	0.18	0.00	0.50	0.31	0.11	0.18
n-Valerate, %	0.00	0.18	1.04	1.97	0.43	0.19	< 0.01	0.37
Iso-Valerate, %	0.27	0.38	0.26	0.00	0.29	0.79	0.49	0.49
Jejunum								
Total SCFA, µmol/g	5.99	7.80	9.23	9.24	1.51	0.52	0.11	0.53
Acetate, %	97.9	83.9	92.9	90.3	6.13	0.18	0.91	0.34
Propionate, %	2.12	7.28	7.15	8.46	4.75	0.48	0.50	0.68
n-Butyrate, %	0.00	4.91	0.01	0.81	1.78	0.11	0.24	0.24
Iso-Butyrate, %	0.00	3.92	0.00	0.00	1.07	0.07	0.07	0.07
n-Valerate, %	0.00	0.00	0.00	0.47	0.22	0.27	0.27	0.27
Iso-Valerate, %	0.00	0.00	0.00	0.00	.	.	.	.
Colon								
Total SCFA, µmol/g	63.3	93.5	67.2	81.2	17.0	< 0.01	< 0.01	0.89
Acetate, %	76.9	75.3	67.1	68.0	1.35	0.78	< 0.01	0.36
Propionate, %	14.6	14.8	19.2	20.6	1.06	0.49	< 0.01	0.58
n-Butyrate, %	4.17	5.98	11.6	10.1	1.08	0.92	< 0.01	0.14
Iso-Butyrate, %	1.48	1.30	0.73	0.49	0.11	0.06	< 0.01	0.76
n-Valerate, %	1.50	1.62	0.74	0.54	0.20	0.84	< 0.01	0.44
Iso-Valerate, %	1.26	1.01	0.59	0.37	0.11	0.04	< 0.01	0.87

^1^Medium-quality hay without concentrate supplementation

^2^High-quality hay without concentrate supplementation

^3^Medium-quality hay with 70% concentrate supplementation (on fresh matter basis)

^4^High-quality hay with 70% concentrate supplementation (on fresh matter basis)

^5^Standard error of the mean

Numbers within a row with different superscript letters indicate difference (*P* < 0.05)

### Host gene expressions along the gastrointestinal tract

**Fig. 5:**
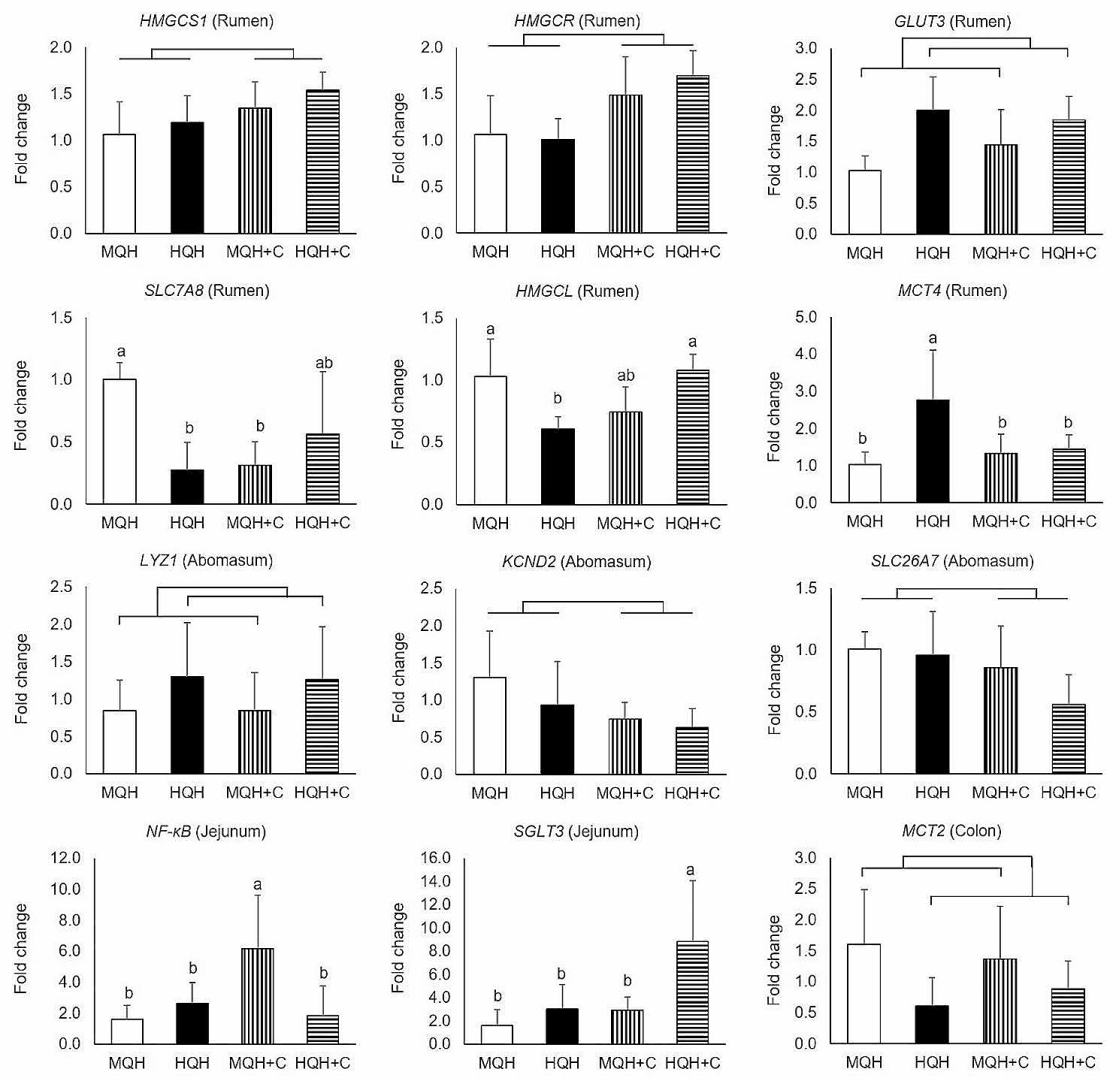
Relative expressions of host genes affected by hay quality, concentrate supplementation or its interaction using MQH treatment as basis. MQH = 100% medium-quality hay; HQH = 100% high-quality hay; MQH + C = 30% medium-quality hay with 70% concentrate (on fresh matter basis); HQH + C = 30% high-quality hay with 70% concentrate (on fresh matter basis)

The relative expressions for all host gene targets in all gut locations can be obtained from Supplementary Table 4 and as shown in Fig. 5, the varying carbohydrate composition in the starter diets influenced the expression of 12 host genes in different locations along the GIT. We observed an interaction of hay quality and concentrate supplementation in both the rumen and the jejunum. For the rumen, expression of *SLC7A8* was approximately fourfold downregulated in both HQH and MQH + C when compared to MQH, whereas HQH + C did not differ from the other treatments (*P* = 0.01). The *HMGCL* expression was lower in HQH than in MQH and HQH + C with MQH + C being intermediate (*P* < 0.01). Besides, HQH feeding resulted in a higher expression of *MCT4* than for other treatment groups (*P* = 0.03). In the jejunum, a threefold upregulation was found for *NF-κB* (*P* = 0.01) when feeding MQH + C diet while *SGLT3* expression (*P* = 0.05) was upregulated around fourfold with HQH + C compared to other treatments. Regarding main effects, the supplementation with concentrate upregulated the expression of two host genes associated with cholesterogenesis in the rumen, i.e., *HMGCS1* (*P* = 0.03) and *HMGCR* (*P* < 0.01). In contrast, *KCND2* (*P* = 0.01) and *SLC26A7* (*P* = 0.04) were both downregulated in the abomasum when concentrate was included in the starter diet. Moreover, when compared to MQH feeding, HQH feeding upregulated the expression of *GLUT3* in the rumen (*P* < 0.01) as well as *LYZ1* in the abomasum (*P* = 0.01), whereas *MCT2* expression in colon (*P* = 0.01) was downregulated with HQH feeding. Besides, no further host gene expressions were affected in these gut locations and we did not find an effect of hay quality, concentrate supplementation or its interaction on the host gene expression in the duodenum (each *P* > 0.10; Supplementary Table 4).

### Correlations

**Fig. 6:**
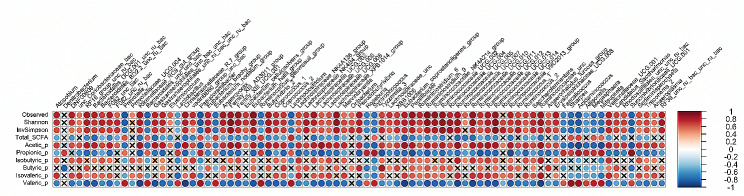
Heatmap illustrating correlations between bacterial genera and alpha diversity indices, SCFA profiles in rumen liquid as well as gene expressions of SCFA transporters in rumen epithelium, considering Spearman correlations with *P* ≤ 0.05 and *r* > 0.70 or *r* < -0.70. Correlations not fulfilling these criteria are indicated by a cross

Our heatmaps based on correlation analysis only included correlations that were considered to be strong, and therefore *r* > 0.70 or *r* < -0.70 [8]. For the rumen liquid (Fig. 6 and Supplementary Fig. 4), only acetate was positively correlated with alpha diversity metrics, whereas both propionate and n-valerate were negatively correlated with all alpha diversity metrics (each *P* < 0.05). Besides, also total SCFA concentration showed a negative correlation with the number of observed sOTU (*P* < 0.05). At the genus level, Family XIII AD3011 group, *Lachnospiraceae* XPB1014 group, *Ruminiclostridium* 9, as well as *Ruminococcaceae* NK4A214 group, *Ruminococcaceae* UGC-002, *Ruminococcaceae* UGC-005, *Ruminococcaceae* UGC-010, *Ruminococcaceae* UGC-013 and an uncultured bacterium belonging to *Ruminococcaceae* had the strongest positive correlation with alpha diversity metrics, i.e., *r* > 0.85, plus positive and negative correlations with acetate and propionate proportions, respectively (each *P* < 0.05). In contrast, no genus showed comparable strong negative correlations, i.e., *r* < -0.85, with alpha diversity metrics, but *Sharpea*, *Megasphaera*, *Prevotella* 7, *Mitsuokella*, *Lachnoclostridium* and *Succinivibrionaceae* UCG − 001 belonged to genera being negatively correlated with alpha diversity (each *P* < 0.05). Likewise, majority of them were negatively and positively correlated to acetate and propionate proportions, respectively (each *P* < 0.05).

**Fig. 7:**
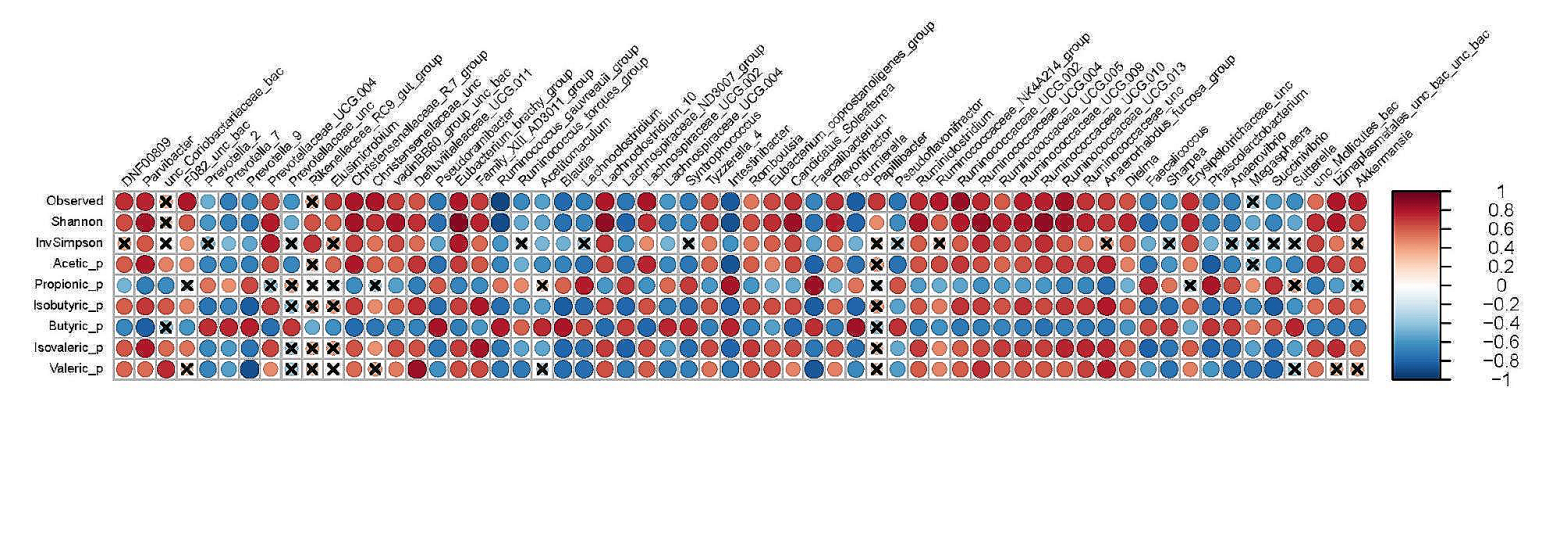
Heatmap illustrating correlations between bacterial genera and alpha diversity indices, SCFA profiles as well as gene expressions of SCFA transporters in the colon, considering Spearman correlations with *P* ≤ 0.05 and *r* > 0.70 or *r* < -0.70. Correlations not fulfilling these criteria are indicated by a cross

For the colon(Fig. 7 and Supplementary Fig. 5), No strong correlations were found for total SCFA concentration and microbial taxa or alpha diversity metrics, whereas proportions of acetate and iso-acids were positively correlated with the number of observed sOTU and Shannon index (each *P* < 0.05). The n-butyrate proportion correlated negatively with these alpha diversity indices, but positively with *Prevotella 7*, which was also negatively related to Shannon diversity and n-valerate (each *P* < 0.05). Strongest positive correlations, i.e., *r* > 0.85, between microbial taxa and alpha diversity were found for *Ruminococcaceae* UCG-002, *Ruminococcaceae* UCG-010, *Ruminococcaceae* UCG-013, *Soleaferrea* and *Lachnoclostridium* 10, whereas *Intestinibacter* and *Ruminococcus gauvreauii* group had strongest negative correlation with alpha diversity metrics, i.e., *r*<-0.85 (each *P* < 0.05).

The relative gene expressions of SCFA transporters were neither strongly correlated with total nor with individual SCFA proportions in both the rumen and colon. Likewise, we obtained no correlation of SCFA transporters with any microbial genus of the rumen liquid or colon.

The additional correlation analysis for relative expressions of host genes associated with keto- and cholesterogenesis in the rumen epithelium and proportions of acetate and n-butyrate in the rumen liquid are presented in Supplementary Table 5. For ketogenesis-associated genes, relative expression of BDH1 correlated positively with ruminal n-butyrate proportion in rumen liquid (*r* = 0.53 and *P* = 0.02). Besides, AACS expression tended to be negatively correlated with n-butyrate proportion (*r*=-0.44 and *P* = 0.06). The relative expression of cholesterogenesis-associated gene HMGCS1 was negatively correlated with n-butyrate proportion in rumen liquid (*r*=-0.56 and *P* = 0.01). Similarly, HMGCR expression was negatively correlated with n-butyrate proportion (*r*=-0.69 and *P* < 0.01) as well as tendentially with acetate proportion in rumen liquid (*r*=-0.39 and *P* = 0.09).

## Discussion

The present study analyzed the microbial communities, SCFA profiles and host epithelial gene expressions along the GIT of fully weaned Holstein calves fed starter diets differing in carbohydrate composition, i.e., hay quality and concentrate supplementation. We observed that concentrate supplementation significantly reduced the microbial diversity and richness in all gut locations. The number of observed sOTU steeply declined with concentrate supplementation, approximately a halving in case of MQH vs. MQH + C and HQH + C. Along with a distinct separation in the beta diversity structure, this indicated a clear differentiation of microbial communities for the entire GIT. Such concentrate-induced drops in microbial diversity are well-described in the fore- and hindgut of adult ruminants [9–11] and derive from the high proportion of starch in the concentrate-supplemented diets, which are efficiently utilized by a certain part of the microbiota that consequently suppress other microbial members. Likewise, concentrate supplementation evoked a substantially smaller CCM that persisted uniformly along the GIT with only a slight weakening towards the hindgut, where the CCM became more diverse than in fore- and midgut – presumably because less starch was entering the hindgut. Indeed, nine genera being present in all major gut regions constituted more than 80% of CCM-genera that were exclusively found in the fore- and midgut of calves fed concentrate-supplemented starter diets. With pure hay feeding, however, we observed the establishment of more diverse CCM that differed between the major gut regions and comprised a large variety of fibrolytic genera, e.g. belonging to *Lachnospiraceae* and *Ruminococcaceae*.

Concentrate supplementation substantially boosted bacterial genera prominently associated with lactate production, like *Lactobacillus* and *Sharpea* [12], and lactate utilization, like *Megasphaera* [13]. These microbial members were further strongly negatively correlated with microbial diversity. Such alterations in the microbial composition were also reflected in higher propionate proportions observed in the forestomach system, abomasum and colon. Similarly, including concentrate in the starter diets upregulated predicted pathways associated with α-glycosidic carbohydrate degradation, whereas functions belonging to the formation of acetate and n-butyrate were reduced. Therefore, the present data clearly support our hypothesis that concentrate supplementation in starter diets prime a microbiota specialized in starch utilization along the full GIT of calves. However, pure hay feeding as well primed the gut ecosystem and led to the establishment of a distinct, albeit less uniform, microbiota along the GIT of calves.

The increased presence of succinate producers in the reticulum and rumen with concentrate supplementation, such as *Succinivibrio* and *Succinivibrionaceae* UCG − 001, as well matched the concept of a microbiota well adapted to the starch-rich starter diet. These succinate producers should have contributed to the propionate increment in those gut locations as around 40% of ruminal propionate typically originates from succinate metabolization [14]. Besides propionate, concentrate supplementation also increased n-valerate levels in all gut locations except the jejunum and colon, therefore, together with the findings on propionate, confirming our hypothesis of a higher provision with glucogenic precursors to calves of the MQH + C and HQH + C groups.

At the host gene expression level, however, concentrate supplementation did not affect SCFA and glucose transporters or inflammation-associated genes. Likewise, SCFA transporters did not strongly correlate with total or individual SCFA in the rumen and colon. Only the gene expression of the glucose transporter *SGLT3* was significantly upregulated for HQH + C compared to other treatments in the jejunum. This may be due to a higher post-ruminal flow of starch into the small intestine than with other starter diets and would match the findings of Zhang et al. [15], who also observed an increased *SGLT3* gene expression in the small intestine of cows receiving rumen-protected glucose.

In terms of host gene expression, we hypothesized an upregulation of genes involved in ketogenesis in the rumen of purely hay-fed calves as those showed substantially higher serum BHB concentrations than concentrate-fed calves, which was putatively related to higher ruminal n-butyrate concentrations and not energy mobilization of calves [1]. Surprisingly, concentrate supplementation had no effect on the expression of any of those genes associated with ketogenesis. As *HMGCS2* represents the key gene initializing ketogenesis [16], the expression of which was not different between treatments, it can explain why also other genes involved in the pathway downstream were not affected. On the other hand, it should be considered that gene expression and actual protein abundance can be modestly correlated [17], meaning that a higher ketogenic activity in the rumen epithelium would not be clearly reproduced at gene expression level.

Calves receiving starter diets with concentrate showed higher expression of both cholesterogenesis-associated genes in the rumen than calves fed purely hay-based diets, therefore indicating an upregulated cholesterol biosynthesis with starch inclusion. However, irrespectively of hay quality, serum cholesterol concentrations were higher in calves fed hay than in calves fed hay and concentrate, which earlier was speculated to be caused by higher ruminal acetate concentrations [1], i.e., an important cholesterol precursor [18], in calves of the MQH and HQH groups. Interestingly, the gene expressions of *HMGCS1* and *HMGCR* were negatively correlated with acetate and n-butyrate proportions in the rumen, which contradicts this assumption. Indeed, Steele et al. [19] described a downregulation of genes associated with cholesterol formation in the rumen epithelium of acidotic dairy cows. But neither calves from MQH + C nor from HQH + C showed signs of rumen acidosis and remained a ruminal pH > 6.0 during the complete experiment [2]. On the other hand, the presence of a negative feedback loop may be considered since cholesterol accumulation in cells reduces the mRNA production of responsible genes, and vice versa [20]. Therefore, the higher ruminal *HMGCS1* and *HMGCR* gene expressions that we observed with concentrate supplementation would match the lower cholesterol concentrations in the blood.

Regarding the small intestine of calves, a decline in microbial diversity due to concentrate supplementation has not been described before and concentrate inclusion decreased prominent fibrolytic bacteria, such as *Ruminococcaceae* UCG-013 or members of *Lachnospiraceae*, whereas various fibrolytic genera were part of the CCM in calves without concentrate supplementation. Generally, the sections of the small intestine showed a lower microbial diversity compared to the rumen or colon, which is in line with previous findings in adult ruminants [11, 21, 22] and indicates the presence of fewer but well-adapted microbes that can cope with the specific conditions of the small intestine, i.e., high passage rate, nutrient competition with host enzymes and digestion secretes [21]. However, it needs to be noted that concentrate supplementation reshaped both the fermentation pattern and the structure of the microbiota in the small intestine in a similar fashion as in the other gut locations.

High-concentrate feeding regimes are also well-known to trigger the emergence of local inflammations in the bovine gut, especially in the rumen and hindgut [23, 24]. For the small intestine, only very sparse information is yet available. Our host gene expression analysis revealed a substantial upregulation of pro-inflammatory *NF-κB* in the jejunum of MQH + C group, thus providing first indication that also the bovine small intestine might be negatively affected by high-concentrate diets. Thereby, the jejunum constituted the only gut location showing a distinctive response of pro-inflammatory genes to dietary treatments. It may be due to the fact that highly immunoresponsive tissue is located in the jejunum, i.e., the Peyer’s patches [25], which consequently caused a first inflammatory signal only in this part of the GIT. However, no inflammatory signs in the fore- and hindgut or at systemic level were observed in any of the calves [2] and a more diverse function of *NF-κB* than solely inducing a pro-inflammatory signaling pathway is often discussed [26]. Likewise, it still remains uncertain why the *NF-κB* gene expression was upregulated in MQH + C calves but not in the HQH + C group. One possible explanation might be that MQH + C calves selected more for concentrates than HQH + C group [1], leading to a higher intake of non-fiber carbohydrates by the MQH + C group (data not published).

Interestingly, concentrate supplementation had a contrasting effect on n-butyrate in the fore- and the hindgut: while concentrate supplementation reduced the n-butyrate proportion by around 50% in the reticulum, rumen fluid and rumen solid, we observed a doubling of its share with concentrate supplementation in the colon. Therefore, our hypothesis of more acetate and n-butyrate with pure hay feeding was not confirmed for the latter SCFA. We assume that the rumen microbes primarily fermented the concentrate, leading to depressed ruminal fiber degradation and eventually lower proportions of n-butyrate and acetate but more propionate in the forestomach system. Consequently, it appears that with the MQH + C and HQH + C diets, fiber fermentation was more shifted towards the hindgut of calves, resulting in higher n-butyrate proportions in the hindgut that, unexpectedly, were associated with a reduced colonic microbial diversity. In this context, we propose that *Prevotella* 9 may be a key cluster as it was most increased and thereby a core genus in the colon of calves with concentrate supplementation. Moreover, our correlation analysis revealed *Prevotella* 9 to be strongly positively correlated with colonic n-butyrate concentration, which is also in line with data from pre-weaned Holstein calves [27]. Besides, a certain proportion of ruminally non-fermented and by the host undigested starch should have also reached the colon as propionate proportion was still considerably higher with concentrate supplementation than without. This was also reflected in a higher fecal pH for pure hay starter diets [2]. Therefore, concentrate supplementation promoted a CCM in the hindgut harboring *Prevotella* 7, *Sharpea*, *Succinivibrio* and *Succinivibrionaceae* UCG − 001, i.e., microbes involved in starch utilization that were not part of the CCM without concentrate supplementation.

Apart from the strong impact of concentrate supplementation that we observed along the entire GIT of the calves, hay quality as well influenced the microbial ecosystem and host epithelial gene expression. Hereby, the effects were predominantly present in the fore- and hindgut, meaning the gut locations with significant fermentative activity. The lower microbial diversity when feeding HQH instead of MQH diets should be caused by the higher contents of sugars and relatively lower fiber concentrations in the high-quality hay, which initiated a microbial phenotype that was differentiated from medium-quality hay as analogously observed for concentrate supplementation – albeit to a much lesser extent. Besides sugars and fiber, the crude protein concentration was as well different between MQH and HQH, i.e., 14.9% vs. 21.0% in DM, and bacteria degrading nitrogenous compounds in the rumen are indeed affected by dietary factors [28]. Still, we could not identify proteolytic members in the CCM or differential abundances along the calves’ gut. The higher sugar provision with HQH was reflected by an upregulated gene expression of SCFA transporter *MCT4* in the rumen as well as the increased proportions of propionate along with lower acetate proportions in reticulum and both rumen fluid and solid. In this context, the reduction of fibrolytic *Ruminococcaceae* UCG-011 and *Lachnospiraceae* FCS020 group in the rumen liquid when feeding HQH instead of MQH diets supports the findings on a microbial composition level. However, it should be noted that concentrate supplementation had a much stronger impact on the microbial ecosystem, which became also clearly apparent by the indeed low level of differentiation of CCM due to distinct hay quality, particularly when compared to the influence of the factor concentrate. Hereby, it is also possible that the factor concentrate has superimposed the impact of different hay qualities [3].

The above described effects of hay quality on propionate proportions in the forestomach system were not present in the colon, likely because rumen microbes have directly utilized the greater sugar content coming from the HQH diet. Therefore, the impact of hay quality on the microbial ecosystem along the GIT was less lasting compared to concentrate supplementation. However, colonic iso-butyrate and iso-valerate proportions were higher with MQH than with HQH diets, which may rather originate from protein than carbohydrate fermentation [28]. Despite its lower crude protein concentration when compared to high-quality hay, the medium-quality hay was harvested at a later stage of maturity [1] and therefore could have contained more protein with a slow ruminal degradability [29], consequently escaping the rumen to a larger part and being fermented in the colon – especially as such fiber-bound protein is not available for host digestion in the small intestine. Indeed, the genera *Parvibacter* and *Romboutsia*, both harboring iso-butyrate and iso-valerate producers [30, 31], were positively correlated with iso-acid proportions in the colon and *Romboutsia* was part of the hindgut CCM for medium-quality hay while it was not for high-quality hay. Likewise, the elevated abundance of major fibrolytic clusters in the CCM of the hindgut with pure hay feeding may not only be caused by the shift of fiber degradation from fore- to hindgut, but in part also by the higher levels of iso-acids, which stimulate the growth of fibrolytics [32].

## Conclusions

The carbohydrate composition of starter diets substantially shapes the microbial ecosystem, meaning composition and activity, along the entire GIT of Holstein calves. Compared to pure hay diets, concentrate supplementation drastically reduced microbial diversity in all gut sites, including the small intestine, with a narrow and distinct CCM dominated by *Prevotella* and increments in lactate producers and utilizers. Therefore, concentrate supplementation primed the microbial communities on the efficient utilization of starch components along the full GIT. Likewise, the altered SCFA profile revealed lower acetate and higher propionate proportions in response to concentrate supplementation along the GIT, whereas concentrate supplementation decreased the proportion of n-butyrate in the foregut but increased it in the colon. Regarding hay quality, predominantly the fore- and hindgut, i.e., gut locations with substantial fermentative activity, responded to this dietary factor with lower microbial diversity with HQH feeding as well as altered abundances of key fibrolytics and SCFA patterns in the rumen. The hay quality, however, had a less pronounced priming force compared to concentrate supplementation and was mainly restricted to the forestomach system. The concentrate-rich starter diets caused no changes in the expression of inflammation-related genes along the calves’ gut. Only *NF-κB* gene expression was increased in the jejunum, suggesting to include the small intestine into future gut health-related research. Regarding higher serum BHB concentrations in purely hay-fed calves, however, gene expression analysis provided no answer, as we observed no upregulation of genes involved in ketogenesis in the rumen. Therefore, other mechanisms must have been responsible, which is also true for cholesterol biosynthesis and demands for investigation in further studies.

### Methods

This study was conducted under the national authority according to § 26 of the Law for Animal Experiments, Tierversuchsgesetz 2012-TVG (GZ: BMBWF-66.019/0016-V/3b/2019).

### Animals and diets

Details on the experimental setup of the trial as well as housing and feeding of the animals can be obtained from the companion publication Terler et al. [1]. Briefly, the experiment was conducted in a 2 × 2 factorial design with two hay qualities, that is medium- or high-quality hay, and either without or with 70% concentrate inclusion (on fresh matter basis), that is 0% or 40% starch inclusion on a DM basis. After birth, 40 Holstein calves (20 male and 20 female calves) were allocated to four treatment groups (each *n* = 10): (i) 100% medium-quality hay (MQH), (ii) 100% high-quality hay (HQH), (iii) 30% MQH and 70% concentrate (on fresh matter basis; MQH + C), (iv) 30% HQH and 70% concentrate (on fresh matter basis; HQH + C). These treatment diets were offered to the calves ad libitum during the complete experiment and the chemical composition of the feedstuffs can be obtained from Table 5. Besides the four different solid feeds, all calves were offered acidified raw whole milk according to a standard milk feeding plan with ad libitum access for the first four weeks and a subsequently gradual reduction of milk allowance until calves were completely weaned at the end of week 12. Two calves had to be excluded before the end of the experiment due to health issues and were therefore not considered in data analysis, i.e., one female calf of the MQH group and one male calf of the MQH + C group.

**Table 5:** Chemical composition of feedstuffs fed to Holstein calves (Terler et al. [1])

Item	Medium quality hay	High quality hay	Concentrate
Dry matter (g/kg)	899 ± 24	877 ± 30	891 ± 13
Crude protein (g/kg DM)	149 ± 29	210 ± 11	193 ± 9
Ether extract (g/kg DM)	18 ± 3	24 ± 3	18 ± 2
Ash (g/kg DM)	76 ± 7	86 ± 3	39 ± 11
NDF^1^ (g/kg DM)	522 ± 24	455 ± 15	204 ± 12
ADF^2^ (g/kg DM)	329 ± 15	247 ± 11	66 ± 5
ADL^3^ (g/kg DM)	49 ± 7	23 ± 3	13 ± 2
NFC^4^ (g/kg DM)	235 ± 34	225 ± 16	547 ± 16
WSC^5^ (g/kg DM)	124 ± 34	205 ± 10	-
ME^6^ (MJ/kg DM)	9.4 ± 0.4	11.2 ± 0.2	13.5 ± 0.2
peNDF_> 8mm_^7^ (% DM)	38.0 ± 6.3	43.1 ± 0.5	-

^1^NDF = Neutral detergent fiber

^2^ADF = Acid detergent fiber

^3^ADL = Acid detergent lignin

^4^NFC = Non-fiber carbohydrates = (1,000 – ash – crude protein – ether extract – NDF)

^5^WSC = Water-soluble carbohydrates

^6^ME = Metabolizable energy

^7^peNDF>8 mm = physically effective NDF > 8 mm

### Sample collection

Seventeen male and three female calves with an average body weight of 128.8 kg ± 19.8 kg were slaughtered at an age of 100 ± 4 d, meaning 5 calves from each treatment group. The initial experimental plan foresaw the slaughtering of only male calves in order to keep female calves for replacement in the dairy herd. However, due to an opposite-sex twin birth plus the exclusion of a male calf from the experiment, three female calves were included in this investigation in order to study calves representing the average of each treatment. Thus, treatment groups MQH, MQH + C and HQH + C included a female calf, while the HQH group did only consist of male calves. The slaughtering process has been described in detail in Terler et al. [33]. Briefly, animals had access to feed until 17:00 h at the day before slaughter and to drinking water until slaughter at 08:00 h in the morning. Then, each calf was stunned with a captive bolt gun and killed by bleeding using throat cutting. Directly after death was ascertained, the abdominal cavity was opened and the GIT was excised and spread. Subsequently, digesta samples from six different gut sites were collected, i.e., reticulum, rumen, abomasum, duodenum, jejunum and colon. The rumen content was additionally separated into rumen liquid and solid digesta for SCFA and microbiota composition analyses. Aliquots for SCFA analysis and microbial community characterization were placed in 2 ml cryotubes (Sarstedt AG & Co. KG, Nümbrecht, Germany) and snap frozen in liquid nitrogen before being stored at −20 °C and − 80 °C for analyses of SCFA and microbial communities, respectively.

After digesta samples had been collected, tissue samples of the respective gut sites were collected, i.e., tissue for the rumen was sampled caudal from the reticulum in the ventral area of the rumen, for the abomasum in the middle of the fundus, for duodenum approximately 10 cm from pylorus, for jejunum approximately 100 cm from pylorus and for colon approximately 50 cm from the exit of the caecum. At first, each gut tissue was rinsed with ice-cold 1x PBS buffer (pH 7.4) to remove blood and digesta particles and then cut into pieces of 2 mm × 2 mm. Subsequently, the cut tissues were snap frozen liquid nitrogen and placed in 2 ml cryotubes (Sarstedt AG & Co. KG, Nümbrecht, Germany) before being stored at -80 °C until RNA extraction.

### DNA extraction and sequencing

DNA was extracted from digesta samples using the DNeasy PowerSoil Kit (Qiagen, Hilden, Germany) as described in Pacífico et al. [10]. Briefly, approximately 250 mg of digesta was placed in a bead beating tube, mixed with solution C1 and then incubated at 95 °C for 5 min. Samples were centrifuged, and the supernatant was collected in a fresh tube and placed on ice. In the meantime, the pellet was mixed with 100 µl of 100 mg/ml lysozyme and 10 µl of 2.5 U/ml mutanolysin (Sigma-Aldrich Co. LLC., USA) and incubated at 37 °C for 30 min. Then, 21.3 µl of 18.8 mg/ml proteinase K (Sigma-Aldrich Co. LLC., USA) was added and again incubated at 37 °C for 60 min. Pellets were placed in a FastPrep-24 device (MP Biomedicals, Santa Ana, USA) for bead beating and supernatant was collected after centrifugation. Cell debris and PCR inhibitors were removed through several centrifugation steps using the solutions C2–C5 provided by the kit. Finally, the supernatant was transferred to a fresh tube and DNA was eluted in 100 µl of C6 buffer. After isolation, total DNA quantity was determined in the Qubit Fluorometer 4.0 (Life Technologies Corporation, Carlsbad, USA) with the Qubit dsDNA HS Assay Kit (Thermo Fisher Scientific, Vienna, Austria) according to the manufacturer’s instructions. All DNA extracts were stored at −20 °C until 16 S rRNA gene amplicon sequencing. The 16 S rRNA gene amplicon sequencing was performed using the NovaSeq 6000 sequencing platform (Novogene Co., Ltd, Cambridge, United Kingdom) and the hypervariable region V3–V4 of bacterial 16 S rRNA gene (2 × 250 bp) were amplified using the primer pair 341 F (5′-CCTAYGGGRBGCASCAG-3′) and 806R (5′-GGACTACNNGGGTATCTAAT-3′) from Yu et al. [34]. Primers were trimmed and corresponding overlapping paired-end reads were stitched by Novogene (Novogene Co., Ltd, Cambridge, United Kingdom).

### Short-chain fatty acid analysis

The sample preparation and analysis of SCFA concentrations were performed as described in Poier et al. [2]. In brief, digesta samples were thawed overnight in the fridge (4 °C) and mixed thoroughly. Subsequently, 1 g of digesta was diluted in 1 mL of distilled water and 300 µl of internal standard 4-methylvaleric acid (Sigma-Aldrich Co. LLC., USA) and 200 µl of 25% phosphoric acid (v/v) were added. Mixtures were vortexed vigorously and centrifuged for 20 min at 20,000 × g and 4 °C. Afterwards, supernatants were transferred into fresh tubes and again centrifuged until being clear. The SCFA concentrations were determined in a gas chromatography device (Shimadzu GC Plus with FID detector) equipped with a 30 m × 0.53 mm i.d. × 0.53 μm capillary column (Trace TR Wax, Thermo Fisher Scientific, Vienna, Austria). The temperatures of injector and detector were 200 and 220 °C, respectively, and helium was used as carrier gas with a flow rate of 1 mL/min.

### Host gene expression analysis

Total RNA was extracted from tissue samples using the RNeasy Mini Qiacube Kit (Qiagen, Hilden, Germany). In brief, approximately 30 mg of tissue was disrupted and homogenized in 350 µl lysis buffer using 600 mg of ceramic beads with 1.4 mm diameter (VWR International GmbH, Vienna, Austria). The RNA was eluted in 50 µl of RNase-free water and genomic DNA was digested with DNAse I (Ambion® TURBO DNA-free™ Kit; Thermo Fisher Scientific, Vienna, Austria). Subsequently, quantity and quality of RNA were assessed on the Qubit Fluorometer 4.0 (Life Technologies Corporation, Carlsbad, USA) using the Qubit RNA HS Assay Kit and the Qubit RNA IQ Assay Kit (Thermo Fisher Scientific, Vienna, Austria). All samples had a RNA integrity number > 8.0. Subsequently, reverse transcriptase quantitative polymerase chain reaction (RT-qPCR) analysis was performed. Firstly, complementary DNA (cDNA) was synthesized from 2 µg of total RNA for rumen and abomasum tissue or 1 µg of total RNA for tissue from duodenum, jejunum and colon using the High Capacity cDNA RT kit (Life Technologies Limited, Vienna, Austria) and a RNase Inhibitor (40,000 U/mL, Biozym Biotech Trading GmbH, Vienna, Austria). Additionally, minus reverse transcription controls were included to monitor for residual DNA contamination. This step was conducted on a thermocycler (Nexus, Eppendorf, Hamburg, Germany) according to the following protocol: 25 °C for 10 min, 37 °C for 2 h, 85 °C for 5 min and a final cooling down at 4 °C. The RT-qPCR were then performed on a C1000 Touch thermocycler equipped with a CFX96 Real-Time System (Bio-Rad Laboratories Inc., Hercules, USA) and included no template controls and minus reverse transcription controls. Each reaction was performed in duplicate with a total volume of 10 µl, including 5 µL mastermix (Blue S’Green, Biozym Biotech Trading GmbH, Vienna, Austria), 20 ng cDNA, 80 nM of respective forward and reverse primer and the following amplification conditions: 3 min at 95 °C, 40 cycles for 5 s at 95 °C and 30 s at the respective annealing temperature for each gene target (Supplementary Table 6), and a final melting curve analysis to ensure primer specificity.

After RT-qPCR, the relative gene expressions were calculated using the ΔΔCt method with MQH treatment as reference [35]. The genes of nuclear factor kappa-B (*NF-κB*) and tumor necrosis factor-alpha (*TNFα*), both involved in the pro-inflammatory immune response, were determined in all tissue samples. The genes encoding for isomers 1, 2 and 4 of monocarboxylate transporter (*MCT1*, *MCT2*, *MCT4*), all involved in transepithelial SCFA transport, were determined in the rumen and colon. The genes encoding for sodium-dependent glucose transporter 3 (*SGLT3*), glucose transporter 3 (*GLUT3*) and solute carrier family 7 member 8 (*SLC7A8*), all three representing glucose transporters, were determined in the rumen, duodenum and jejunum. The genes acetoacetyl-CoA synthetase (*AACS*), acetyl-CoA acetyltransferase 1 (*ACAT1*), 3-hydroxy-3-methylglutaryl CoA synthase 2 (HMGCS2), 3-hydroxy-3-methylglutaryl-CoA lyase (*HMGCL*) and 3-hydroxybutyrate dehydrogenase 1 (*BDH1*), all involved in ketogenesis, as well as the genes 3-hydroxy-3-methylglutaryl-CoA reductase (*HMGCR*) and 3-hydroxy-3-methylglutaryl CoA synthase (*HMGCS1*), both involved in cholesterogenesis, were determined in the rumen tissue samples. The genes encoding for ATPase H+/K + transporting subunit alpha (*ATP4A*), solute carrier family 26 member 7 (*SLC26A7*), solute carrier family 4 member 2 (*SLC4A2*), potassium voltage-gated channel subfamily D member 2 (*KCND2*) and chloride intracellular channel 6 (*CLIC6*), all involved in secretory processes during host’s digestion, as well as the three genes encoding for lysozyme C (*LYZ1*), pepsinogen 5 group I (pepsinogen A; *PGA5*) and chymosin (*CYM*), all involved in host’s protein digestion, were determined in the abomasal tissue samples. We used three housekeeping genes for all tissue samples to control our samples for mRNA content and β-actin (*ACTB*) and ribosomal protein L19 (*RPL19*) were used for all GIT tissues. Additionally, ornithine decarboxylase antizyme 1 (*OAZ1*) served as third housekeeping gene for the rumen and abomasum tissues, whereas tyrosine 3-monooxygenase/tryptophan 5-monooxygenase activation protein zeta (*YWHAZ*) completed the set of housekeeping genes for the tissues from duodenum, jejunum and colon.

The primer pairs that were newly designed for this study, were designed by exon overlapping based on published bovine sequences (ARS-UCD1.2) in Ensembl [36] using the Primer3 tool [37]. Optimal annealing temperature and amplification product specificity was determined for each primer pair by gradient PCR and melting curve analysis, respectively. All applied primers had an average efficiency above 0.8 as determined according to Zhao and Fernald [38]. The sequences used for the RT-qPCR as well as the respective annealing temperatures, PCR product sizes and accession numbers in the UCSC human genome browser [39] are listed in Supplementary Table 6. Primer pairs were newly designed for this study or taken from literature [40–45].

### Bioinformatic and statistical analyses

All sequencing reads were processed using the software package Quantitative Insights into Microbial Ecology, i.e., QIIME2 v2020.2 [46]. The read quality was inspected using FASTQC for demultiplexed Illumina fastq data with the PHRED score offset of 33. Sequence data was merged with VSEARCH [47] and then quality filtered using the q-score-joined plugin and 20 as a minimum acceptable PHRED score. Denoising into sOTU was obtained using Deblur [48] and representative sequences and feature tables were filtered in order to exclude sOTU that are classified as mitochondria or chloroplast sequences. Likewise, archaeal sOTU were excluded from further analysis as the applied primer pair may result in biased amplification of archaeal 16 S rRNA genes. All resulting filtered sOTU were aligned with mafft [49] and a phylogeny was constructed with FastTree2 [50]. Taxonomy was assigned to sOTU using a classify-sklearn naïve Bayes taxonomy classifier trained with the 341 F/802R primer set against the SILVA 132 99% OTUs reference sequences [51]. For further analysis, the filtered feature table, rooted tree and taxonomy were imported in Rstudio v14.1717. The sequencing depth was determined according to Chao and Jost [52] using the metagMisc package with all samples having a coverage of > 0.99.

The statistical analysis of SCFA, microbial alpha-diversity and host gene expression data was performed in SAS (v. 9.4, SAS Institute Inc., Cary, USA). First, proc univariate was used to test for normal distribution of data and subsequently data was analyzed by proc mixed with gut location, hay quality and concentrate supplementation as fixed effects as well as animal and sex as random effects. Since the variables were different and strongly affected by gut location, which also matches previous research indicating quantitative and qualitative differences in composition and activity of the microbiota between different gut sites in ruminants (e.g. [21]). we continued the analysis with separating the data by gut location. Therefore, fixed effects of hay quality, concentrate supplementation and their interaction was analyzed for each gut location and differences between least square means were determined by Tukey post-hoc test.

For the rumen liquid and colon digesta, heatmaps based on Spearman correlations that were determined using Hmisc package in Rstudio were generated. Thereby, we included the datasets of microbial genera, total SCFA concentration and individual SCFA proportions as well as relative host gene expressions of SCFA transporters MCT1, MCT2 and MCT4 and only considered significant and strong correlations, i.e., *P* ≤ 0.05 and *r* > 0.70 or *r* < -0.70 [8]. Additionally, Spearman correlations only for specific SCFA proportions and relative expressions of host genes associated with keto- and cholesterogenesis in the rumen were calculated using proc corr. Significance was declared at *P* ≤ 0.05 and trends were considered at 0.05 < *P* ≤ 0.10 for all analyses.

For sequencing data, differences in beta-diversity matrices were calculated using the vegan package and the *adonis2* function in Rstudio [53]. Principal Coordinates Analysis (PCoA) plots were created using Aitchison metrics on clr-transformed data. The differential abundance of microbial genera were calculated for the main factors concentrate supplementation and hay quality and for each gut location using the MaAsLin2 package in Rstudio [54]. Changes in abundances of genera were considered as relevant if coefficient was <−2.00 or > 2.00 and Benjamini-Hochberg false discovery rate-adjusted *q*-values ≤ 0.05.

We further analyzed the CCM at genus level in the digesta, which was defined following Risely [55] as the genera with a relative abundance > 1% in the GIT of calves raised with or without concentrate supplementation and with high- or medium-quality hay. Therefore, datasets from each individual gut location were merged into the three major gut regions fore-, mid- and hindgut, i.e., the foregut comprising reticulum, liquid, and solid rumen, the midgut comprising the abomasum, duodenum, and jejunum and the hindgut comprising the colon, and subsequently filtered for genera with a relative abundance > 1% and a prevalence of > 50% per gut region and treatment factor. Then, Venn diagrams were created using Venny v2.1 [56] to determine which CCM-genera were common or exclusive in the three major gut regions of calves raised with or without concentrate supplementation and with high- or medium-quality hay.

In addition, a predicted function analysis was carried out with PICRUSt2 [57] and differential pathway abundances for the main factors concentrate supplementation and hay quality and for each gut location were analyzed using MaAsLin2 [54] with thresholds for relevance of <−2.00 or > 2.00 and ≤ 0.05 for coefficients and Benjamini-Hochberg false discovery rate-adjusted *q*-values, respectively. The predicted pathways with a differential abundance were matched against the MetaCyc database [58].

### Electronic supplementary material

Below is the link to the electronic supplementary material.

Supplementary Material 1

Supplementary Material 2

Supplementary Material 3

Supplementary Material 4

Supplementary Material 5

## Data Availability

Sequences have been submitted to the Sequence Read Archive (SRA) of the National Center for Biotechnology Information (NCBI) under the accession number PRJNA818123.

## Abbreviations

*AACS*: Acetoacetyl-CoA synthetase
*ACAT1*: Acetyl-CoA acetyltransferase 1
*ACTB*: β-actin
ADF: Acid detergent fiber
ADL: Acid detergent lignin
*ATP4A*: ATPase H+/K + transporting subunit alpha
*BDH1*: 3-hydroxybutyrate dehydrogenase 1
BHB: Beta hydroxybutyrate
CCM: Diet-associated common core microbiota
*CLIC6*: Chloride intracellular channel 6
*CYM*: Chymosin
DM: Dry matter
*GLUT3*: Glucose transporter 3
GIT: Gastrointestinal tract
*HMGCL*: 3-hydroxy-3-methylglutaryl-CoA lyase
*HMGCR*: 3-hydroxy-3-methylglutaryl-CoA reductase
*HMGCS1*: 3-hydroxy-3-methylglutaryl CoA synthase
*HMGCS2*: 3-hydroxy-3-methylglutaryl CoA synthase 2
HQH: 100% high-quality hay (Treatment)
HQH + C: 30% high-quality hay with 70% concentrate on fresh matter basis (Treatment)
*KCND2*: Potassium voltage-gated channel subfamily D member 2
*LYZ1*: Lysozyme C
*MCT1*: Isomer 1 of monocarboxylate transporter
*MCT2*: Isomer 2 of monocarboxylate transporter
*MCT4*: Isomer 4 of monocarboxylate transporter
ME: Metabolizable energy
MQH: 100% medium-quality hay (Treatment)
MQH: 30% medium-quality hay with 70% concentrate on fresh matter basis (Treatment)
NDF: Neutral detergent fiber
NFC: Non-fiber carbohydrates
*NF-κB*: nuclear factor kappa-B
*OAZ1*: Ornithine decarboxylase antizyme 1
PCoA: Principal coordinate analysis
peNDF > 8mm: Physically effective NDF > 8 mm
*PGA5*: Pepsinogen 5 group I/pepsinogen A
*RPL19*: Ribosomal protein L19
SEM: Standard error of the mean
*SGLT3*: Sodium-dependent glucose transporter 3
*SLC26A7*: Solute carrier family 26 member 7
*SLC4A2*: Solute carrier family 4 member 2
*SLC7A8*: Solute carrier family 7 member 8
sOTU: Sub-operational taxonomic unit
*TNFα*: Tumor necrosis factor-alpha
WSC: Water-soluble carbohydrates
*YWHAZ*: Tyrosine 3-monooxygenase/tryptophan 5-monooxygenase activation protein zeta
