# Supplementary material for: Dietary carbohydrate sources differently prime the microbial ecosystem but not the epithelial gene expression profile along the complete gut of young calves
Source: Anim Microbiome. 2024 Mar 13;6:12. doi: 10.1186/s42523-024-00297-5 (PMC10935977; doi:10.1186/s42523-024-00297-5)
Supplement: Supplementary file 3 — Supplementary Material 3 [file 42523_2024_297_MOESM3_ESM.docx]

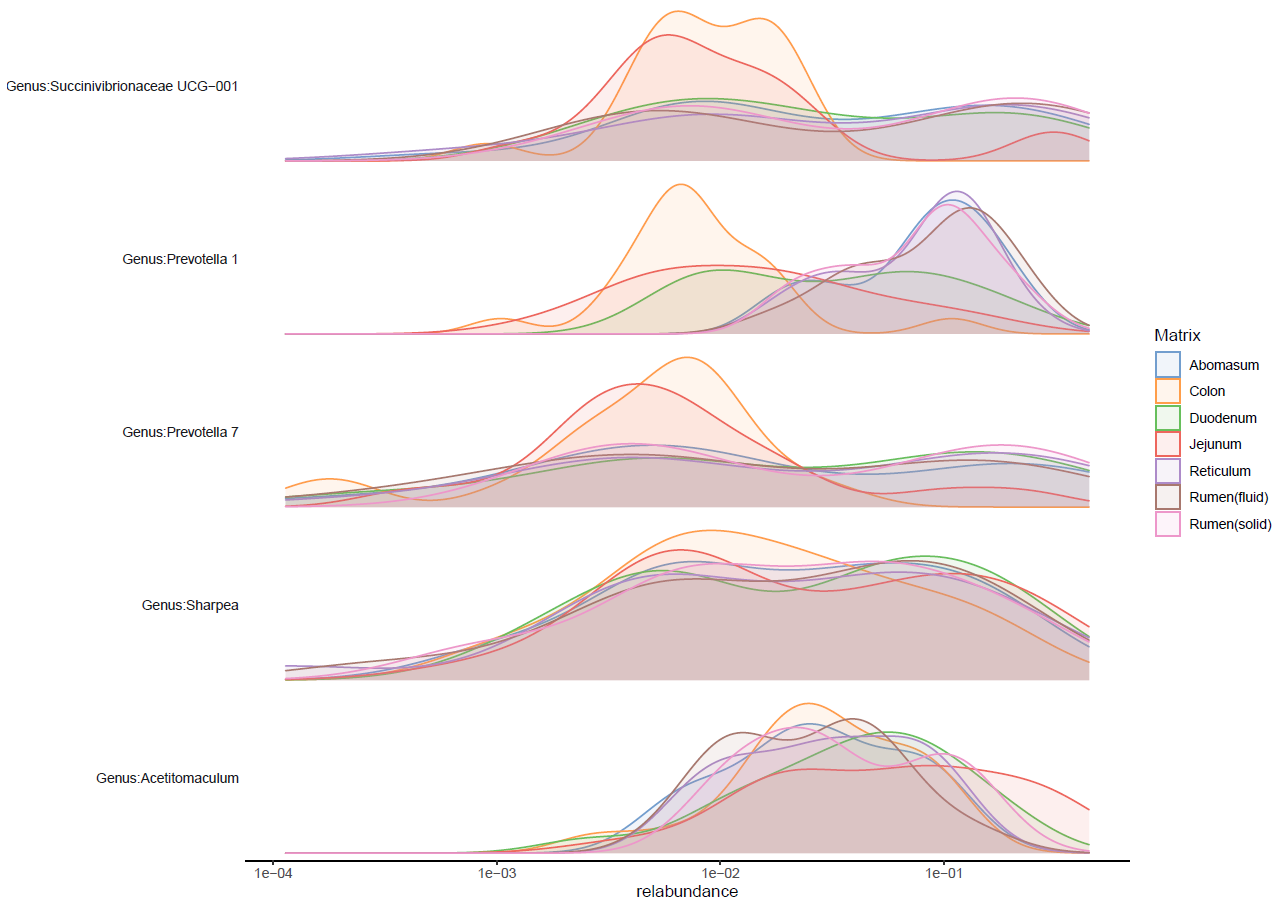


**Supplementary Figure 1.** Abundances of top five most abundant genera across the gastrointestinal tract of calves, irrespectively of fed starter diets.

**
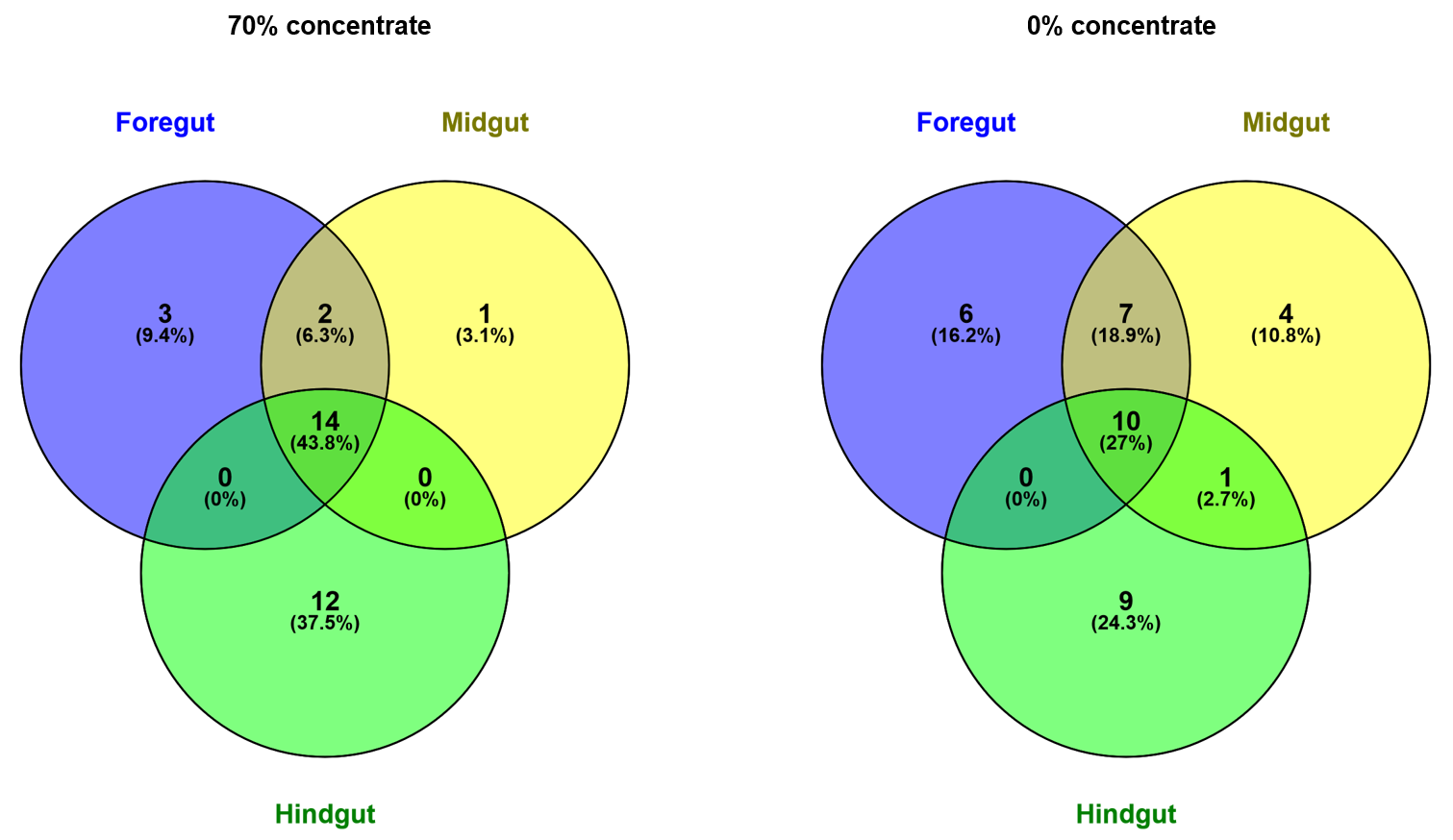
**

**Supplementary Figure 2.** Diet-associated common core microbiota at genus level of the three major gut regions fore-, mid- and hindgut in calves fed starter diets with or without 70% concentrate supplementation (on fresh matter basis).

**
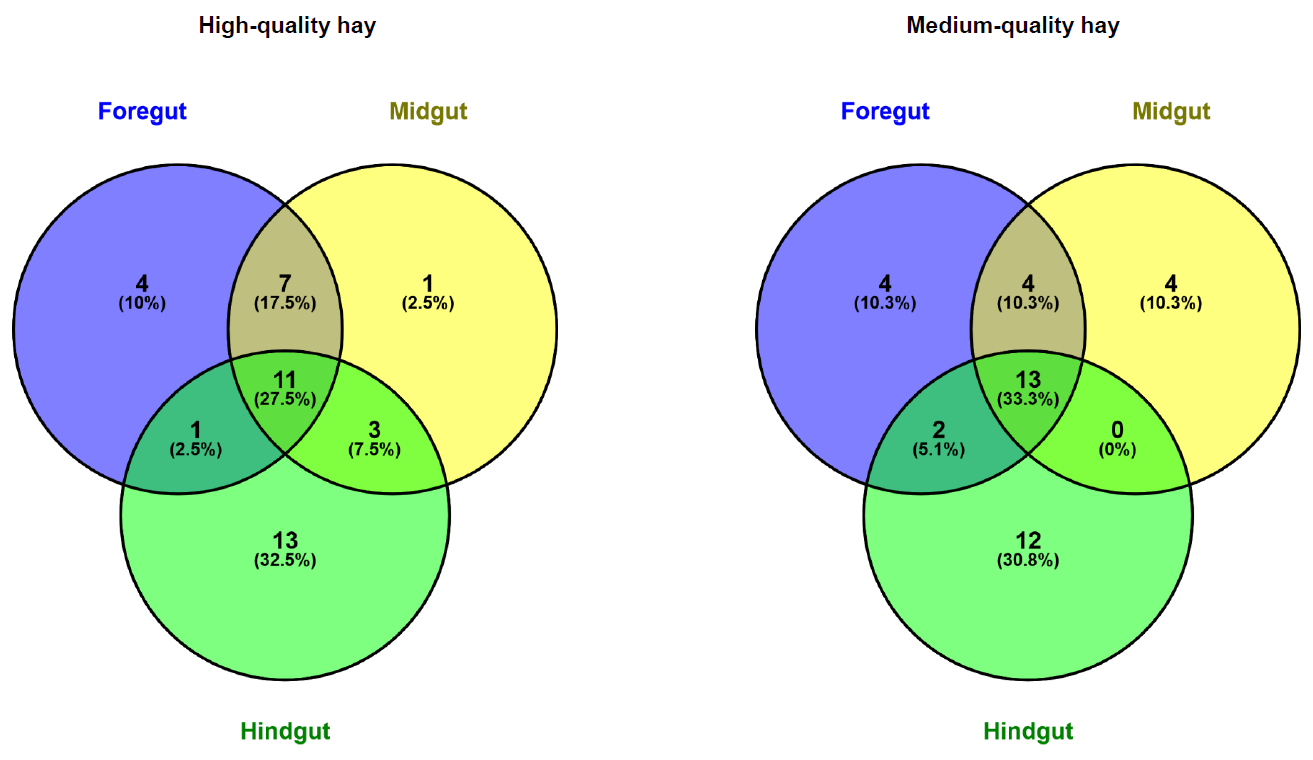
**

**Supplementary Figure 3.** Diet-associated common core microbiota at genus level of the three major gut regions fore-, mid- and hindgut in calves fed starter diets with high- or medium-quality hay.

**
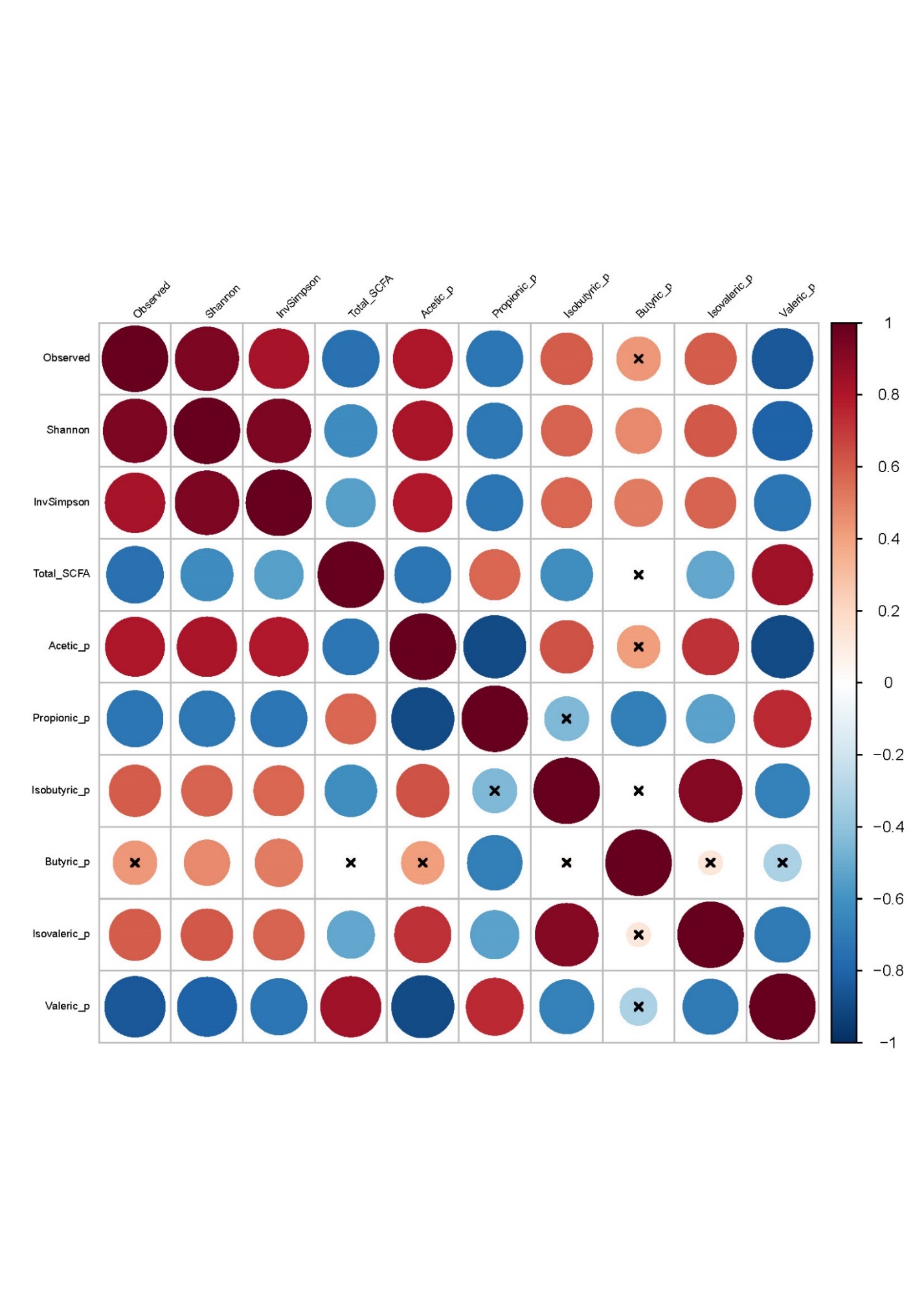
**

**Supplementary Figure 4.** Heatmap illustrating correlations between alpha diversity indices and SCFA profiles in the rumen liquid, considering Spearman correlations with *P* ≤ 0.05 and r > 0.70 or r < -0.70. Correlations not fulfilling these criteria are indicated by a cross.

**
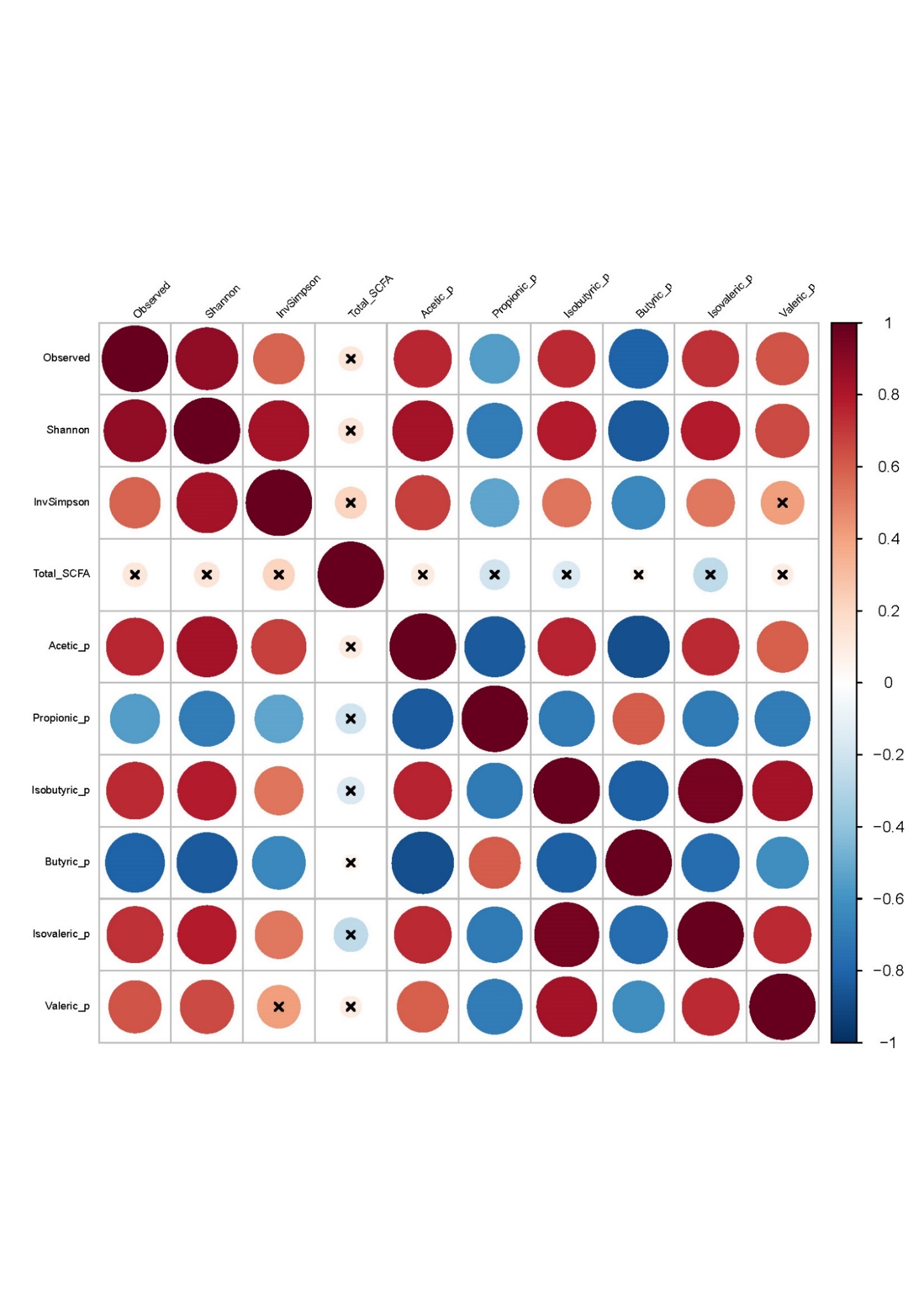
**

**Supplementary Figure 5.** Heatmap illustrating correlations between alpha diversity indices and SCFA profiles in the colon, considering Spearman correlations with *P* ≤ 0.05 and r > 0.70 or r < -0.70. Correlations not fulfilling these criteria are indicated by a cross.
