# Supplementary material for: Dietary carbohydrate sources differently prime the microbial ecosystem but not the epithelial gene expression profile along the complete gut of young calves
Source: Anim Microbiome. 2024 Mar 13;6:12. doi: 10.1186/s42523-024-00297-5 (PMC10935977; doi:10.1186/s42523-024-00297-5)
Supplement: Supplementary file 5 — Supplementary Material 5 [file 42523_2024_297_MOESM5_ESM.docx]

**Supplementary Table 1.** Total read counts and percentage of total read counts at phylum level. Please see the separate Excel-file.

**Supplementary Table 2.** Microbial genera with differential abundance between groups without and with 70% concentrate supplementation (on FM basis) or between distinct hay qualities. Please see the separate Excel-file.

**Supplementary Table 3.** Predicted functional pathways with differential abundance between groups without and with 70% concentrate supplementation (on FM basis) or between distinct hay qualities. Please see the separate Excel-file.

**Supplementary Table 4.** Relative host gene expressions along the gastrointestinal tract of calves fed starter diets differing in carbohydrate composition (MQH treatment as reference).

|  |  | | Treatment group | | | |  | | P-values | | | |  |
| --- | --- | --- | --- | --- | --- | --- | --- | --- | --- | --- | --- | --- | --- |
| # | Matrix | Gene | MQH^1^ | HQH^2^ | MQH+C^3^ | HQH+C^4^ | | SEM^5^ | | Hay | Concentrate | Interaction | |
| 1 | Rumen | *TNFα* | 1.11 | 2.34 | 1.93 | 2.03 | | 0.73 | | 0.37 | 0.73 | 0.44 | |
| 2 | Rumen | *NF-kB* | 1.06 | 1.30 | 0.78 | 1.75 | | 0.40 | | 0.10 | 0.81 | 0.30 | |
| 3 | Rumen | *HMGCS1* | 1.06 | 1.19 | 1.35 | 1.54 | | 0.13 | | 0.23 | 0.03 | 0.80 | |
| 4 | Rumen | *HMGCR* | 1.06 | 1.01 | 1.49 | 1.70 | | 0.15 | | 0.61 | <0.01 | 0.41 | |
| 5 | Rumen | *SGLT3* | 1.18 | 1.50 | 1.22 | 1.63 | | 0.36 | | 0.29 | 0.81 | 0.89 | |
| 6 | Rumen | *GLUT3* | 1.03 | 2.00 | 1.44 | 1.85 | | 0.20 | | <0.01 | 0.53 | 0.18 | |
| 7 | Rumen | *SLC7A8* | 1.01 | 0.28 | 0.31 | 0.57 | | 0.16 | | 0.13 | 0.19 | 0.01 | |
| 8 | Rumen | *HMGCS2* | 1.02 | 0.89 | 0.88 | 1.29 | | 0.16 | | 0.39 | 0.44 | 0.12 | |
| 9 | Rumen | *BDH1* | 1.05 | 0.91 | 0.91 | 0.71 | | 0.17 | | 0.33 | 0.34 | 0.86 | |
| 10 | Rumen | *HMGCL* | 1.03 | 0.61 | 0.75 | 1.08 | | 0.09 | | 0.61 | 0.32 | <0.01 | |
| 11 | Rumen | *AACS* | 1.05 | 1.13 | 1.27 | 1.28 | | 0.14 | | 0.74 | 0.20 | 0.83 | |
| 12 | Rumen | *ACAT1* | 1.02 | 0.74 | 0.70 | 0.85 | | 0.11 | | 0.54 | 0.35 | 0.07 | |
| 13 | Rumen | *MCT2* | 1.05 | 0.92 | 1.09 | 1.36 | | 0.14 | | 0.60 | 0.10 | 0.16 | |
| 14 | Rumen | *MCT1* | 1.15 | 0.85 | 1.17 | 1.04 | | 0.15 | | 0.13 | 0.47 | 0.53 | |
| 15 | Rumen | *MCT4* | 1.04 | 2.76 | 1.34 | 1.44 | | 0.34 | | 0.02 | 0.16 | 0.03 | |
| 16 | Abomasum | *TNFα* | 1.61 | 0.88 | 0.86 | 1.03 | | 0.99 | | 0.74 | 0.72 | 0.59 | |
| 17 | Abomasum | *NF-kB* | 1.05 | 1.92 | 1.91 | 1.89 | | 0.69 | | 0.49 | 0.50 | 0.48 | |
| 18 | Abomasum | *CYM* | 0.86 | 1.28 | 1.18 | 0.95 | | 0.34 | | 0.76 | 0.98 | 0.33 | |
| 19 | Abomasum | *PGA5* | 1.08 | 0.75 | 0.62 | 0.71 | | 0.17 | | 0.48 | 0.15 | 0.22 | |
| 20 | Abomasum | *LYZ1* | 0.84 | 1.30 | 0.85 | 1.26 | | 0.13 | | 0.01 | 0.88 | 0.87 | |
| 21 | Abomasum | *ATP4A2* | 1.04 | 0.97 | 0.77 | 0.78 | | 0.18 | | 0.88 | 0.22 | 0.81 | |
| 22 | Abomasum | *KCND2* | 1.31 | 0.93 | 0.75 | 0.63 | | 0.16 | | 0.12 | 0.01 | 0.39 | |
| 23 | Abomasum | *SLC26A7* | 1.01 | 0.96 | 0.85 | 0.56 | | 0.13 | | 0.19 | 0.04 | 0.34 | |
| 24 | Abomasum | *SLC4A2* | 0.90 | 0.73 | 0.84 | 0.90 | | 0.07 | | 0.40 | 0.42 | 0.10 | |
| 25 | Abomasum | *CLIC6* | 1.01 | 1.03 | 1.18 | 0.84 | | 0.09 | | 0.09 | 0.91 | 0.06 | |
| 26 | Duodenum | *TNFα* | 0.69 | 1.58 | 0.66 | 0.39 | | 0.37 | | 0.38 | 0.10 | 0.11 | |
| 27 | Duodenum | *NF-kB* | 1.20 | 0.91 | 1.42 | 1.61 | | 0.63 | | 0.94 | 0.45 | 0.69 | |
| 28 | Duodenum | *SGLT3* | 1.32 | 1.99 | 1.67 | 2.34 | | 0.57 | | 0.22 | 0.52 | 1.00 | |
| 29 | Duodenum | *GLUT3* | 0.20 | 0.24 | 0.08 | 0.03 | | 0.19 | | 0.95 | 0.34 | 0.79 | |
| 30 | Duodenum | *SLC7A8* | 1.16 | 2.61 | 2.22 | 2.14 | | 0.56 | | 0.22 | 0.59 | 0.17 | |
| 31 | Jejunum | *TNFα* | 0.94 | 0.50 | 0.39 | 2.96 | | 1.51 | | 0.48 | 0.53 | 0.33 | |
| 32 | Jejunum | *NF-kB* | 1.63 | 2.64 | 6.17 | 1.84 | | 0.92 | | 0.06 | 0.03 | 0.01 | |
| 33 | Jejunum | *SGLT3* | 1.59 | 3.06 | 2.91 | 8.81 | | 1.14 | | <0.01 | <0.01 | 0.05 | |
| 34 | Jejunum | *GLUT3* | 0.55 | 0.63 | 0.51 | 1.13 | | 0.55 | | 0.49 | 0.64 | 0.58 | |
| 35 | Jejunum | *SLC7A8* | 0.80 | 1.33 | 1.23 | 1.78 | | 0.49 | | 0.24 | 0.33 | 0.98 | |
| 36 | Colon | *TNFα* | 1.41 | 0.97 | 1.37 | 0.29 | | 0.53 | | 0.12 | 0.44 | 0.50 | |
| 37 | Colon | *NF-kB* | 0.78 | 2.68 | 2.49 | 2.06 | | 0.79 | | 0.34 | 0.48 | 0.15 | |
| 38 | Colon | *MCT2* | 1.60 | 0.61 | 1.36 | 0.89 | | 0.21 | | 0.01 | 0.93 | 0.24 | |
| 39 | Colon | *MCT1* | 0.86 | 0.63 | 0.96 | 0.63 | | 0.24 | | 0.26 | 0.84 | 0.83 | |
| 40 | Colon | *MCT4* | 1.09 | 2.13 | 1.38 | 2.01 | | 0.45 | | 0.08 | 0.85 | 0.65 | |

^1^Medium-quality hay without concentrate supplementation; ^2^High-quality hay without concentrate supplementation; ^3^Medium-quality hay with 70% concentrate supplementation (on fresh matter basis); ^4^High-quality hay with 70% concentrate supplementation (on fresh matter basis); ^5^Standard error of the mean.

**Supplementary Table 5.** Spearman correlation coefficients for relative expressions of host genes associated with keto- and cholesterogenesis in the rumen epithelium and proportions of acetate and n-butyrate in the rumen liquid.

| Host gene | Item | Acetate | n-Butyrate |
| --- | --- | --- | --- |
| *HMGCS2* | coefficient | -0.03 | -0.12 |
|  | P-value | 0.90 | 0.63 |
| *BDH1* | coefficient | 0.12 | 0.53 |
|  | P-value | 0.62 | 0.02 |
| *HMGCL* | coefficient | -0.06 | -0.39 |
|  | P-value | 0.79 | 0.10 |
| *AACS* | coefficient | 0.01 | -0.44 |
|  | P-value | 0.98 | 0.06 |
| *ACAT1* | coefficient | 0.15 | -0.02 |
|  | P-value | 0.54 | 0.94 |
| *HMGCS1* | coefficient | -0.35 | -0.56 |
|  | P-value | 0.15 | 0.01 |
| *HMGCR* | coefficient | -0.39 | -0.69 |
|  | P-value | 0.09 | <0.01 |

**Supplementary Table 6.** Primers used for host epithelial gene expression analysis.

| Target | Item | Oligonucleotide sequence (5‘-3‘) | Product size (bp) | Annealing temperature (°C) | Accession number | Reference |
| --- | --- | --- | --- | --- | --- | --- |
| *ACTB* | Forward | CGTGAGAAGATGACCCAGATCA | 125 | 60 | NM_173979.3 | Steele et al. |
|  | Reverse | TCACCGGAGTCCATCACGAT |  |  |  |  |
| *RPL19* | Forward | AGCCTGTGACTGTCCATTCC | 126 | 60 | ENSBTAG00000002060 | Our study |
|  | Reverse | AGGTTACCTTCTCGGGCATT |  |  |  |  |
| *OAZ1* | Forward | CACAAGAACCGTGATGATCGA | 69 | 64 | NM_001127243.2 | Petri et al. |
|  | Reverse | TCTCACAATCTCAAAGCCCAAA |  |  |  |  |
| *YWHAZ* | Forward | TGAAAGGAGACTACTACCGCTACTTG | 121 | 60 | NM_174814.2 | Petri et al. |
|  | Reverse | GCTGTGACTGGTCCACAATCC |  |  |  |  |
| *TNFα* | Forward | AGCCCTCTRGTTCARACACT | 159 | 63 | ENSBTAG00000025471 | Our study |
|  | Reverse | GCTGGTTGTCTTCCAGCTTC |  |  |  |  |
| *NF-κB* | Forward | ATACGTCGGCCGTGTCTAT | 144 | 58 | NM_001076409.1 | Jin et al. |
|  | Reverse | GGAACTGTGATCCGTGTAG |  |  |  |  |
| *HMGCS1* | Forward | GCTCCGAGAGGATACTCATCAC | 123 | 60 | NM_001206578.1 | Neubauer et al. |
|  | Reverse | CGCCGAGCGTAAGTTCTTCT |  |  |  |  |
| *HMGCS2* | Forward | TCTGGCCCATCACTCTGCC | 126 | 60 | NM_001045883.1 | Van Dorland et al. |
|  | Reverse | AGTGGGGAGCCTGGAGAAGC |  |  |  |  |
| *HMGCR* | Forward | CTAGCCAAGTTCGCCCTCAG | 187 | 60 | ENSBTAT00000010315.5 | Our study |
|  | Reverse | GAACCGACATGCAGCCAAAG |  |  |  |  |
| *HMGCL* | Forward | GGTACGTTCCCAAAGCAAGTG | 248 | 60 | ENSBTAT00000029103.4 | Our study |
|  | Reverse | TTTGGGGTCAGGACTGGGTA |  |  |  |  |
| *ACAT1* | Forward | CAAGGAGGTGAAGGACAGGC | 166 | 64 | ENSBTAG00000012885 | Our study |
|  | Reverse | CTGCCACCATCACATCCTGA |  |  |  |  |
| *BDH1* | Forward | ACTGTCCAGCTCAACGTCTG | 175 | 60 | ENSBTAT00000000573 | Our study |
|  | Reverse | CTTCCGCCACCTCCTTGTAG |  |  |  |  |
| *Target* | Item | Oligonucleotide sequence (5‘-3‘) | Product size (bp) | Annealing temperature (°C) | Accession number | Reference |
| *AACS* | Forward | GGAAGGCAAGGAGGAGATCG | 167 | 60 | ENSBTAT00000007459.6 | Our study |
|  | Reverse | GCCATTCACGCCAAAGTCTG |  |  |  |  |
| *MCT1* | Forward | CTCACCACAGGGGTCCTTAC | 144 | 60 | NM_001037319.1 | Metzler‐Zebeli et al. |
|  | Reverse | AAGTAGCGGTTGAGCATGATGA |  |  |  |  |
| *MCT2* | Forward | GTGCTGTCCTGTCCTCCTTG | 187 | 60 | ENSBTAT00000083837 | Our study |
|  | Reverse | GTGACAGCTTTGGGAAACGC |  |  |  |  |
| *MCT4* | Forward | CATCATGTTGGCTGTCATGTATGG | 77 | 57.5 | NM_001109980.1 | Metzler‐Zebeli et al. |
|  | Reverse | TCCTGCACAGTGTTACAGAAGGA |  |  |  |  |
| *SGLT3* | Forward | CGAAATAGTACAGAGGAACGAATTGA | 114 | 60 | NM_001205942.1 | Our study |
|  | Reverse | CTTCCTGAGACATCCACGAGTTT |  |  |  |  |
| *GLUT3* | Forward | ATGTCGCAGGAGAAGCAAGT | 223 | 64 | ENSBTAG00000004556 | Our study |
|  | Reverse | AGACACCACGGTGAAGATG |  |  |  |  |
| *SLC7A8* | Forward | TTTCGGAGGTTGAAATCCTG | 194 | 60 | ENSBTAT00000009302.5 | Our study |
|  | Reverse | GATTCCAGAGCCGATGATGT |  |  |  |  |
| *ATP4A* | Forward | GAGATGTGCCAGATTGCCGA | 155 | 60 | ENSBTAT00000011569.6 | Our study |
|  | Reverse | CCACACACAGCAAAGGGAAC |  |  |  |  |
| *KCND2* | Forward | ATCGCTCTACCTGTCCCAGT | 168 | 62 | ENSBTAT00000045500.4 | Our study |
|  | Reverse | GAGTAAGCCGTTCCGTTTGC |  |  |  |  |
| *SLC26A7* | Forward | AACCTCACCACACACAGCAA | 184 | 62 | ENSBTAT00000045797.4 | Our study |
|  | Reverse | CCCCAGTTGTCATTGCACTG |  |  |  |  |
| *SLC4A2* | Forward | AAGGCAGAAACCACCAGTCC | 225 | 60 | ENSBTAT00000083376.1 | Our study |
|  | Reverse | GCTTCCAATGCGTCTCCTCT |  |  |  |  |
| *CLIC6* | Forward | TGCCCATTTTCTCAGCGTCT | 239 | 60 | ENSBTAT00000002299.5 | Our study |
|  | Reverse | AGATTCAGGGTGTTGGGTTCC |  |  |  |  |
| *CYM* | Forward | TCGACCCGTCCTACTACACA | 219 | 60 | ENSBTAT00000013970.4 | Our study |
|  | Reverse | CGTACTGGTTCTGTGTGGCT |  |  |  |  |
| Target | Item | Oligonucleotide sequence (5‘-3‘) | Product size (bp) | Annealing temperature (°C) | Accession number | Reference |
| *PGA5* | Forward | GTGCCCTCCATCTACTGCTC | 189 | 60 | ENSBTAG00000014761 | Our study |
|  | Reverse | GCCAAAGATCTGGTTGGTGT |  |  |  |  |
